# Structural, Densimetric, and Thermal Properties of Biodiesel and Diesel Blends

**DOI:** 10.1021/acsomega.5c09853

**Published:** 2025-12-17

**Authors:** Silvania Lanfredi, Tulio B. Araújo, Fabiano R. Praxedes, Paulo M. O. Silva, Fabricio R. Sensato, Marcos A. L. Nobre

**Affiliations:** † Department of Chemistry and Biochemistry, School of Technology and Sciences, 28108 São Paulo State University (Unesp), R. Roberto Simonsen—305, Presidente Prudente, São Paulo CEP 19060-900, Brazil; ‡ Institute of Biosciences, Humanities and Exact Sciences, São Paulo State University (Unesp), São José do Rio Preto, São Paulo 15054-000, Brazil; § Department of Physics, Telecommunication and Materials Science and Engineering Laboratory (LOCEM), Federal University of Ceará (UFC), Pici Campus, Fortaleza, Ceará CEP 60455-760, Brazil; ∥ Department of Exact and Earth Sciences, 505146 Federal University of São Paulo (Unifesp) Diadema Campus, Diadema, São Paulo CEP 09972-270, Brazil; ⊥ Department of Physics, School of Technology and Sciences, São Paulo State University (Unesp), R. Roberto Simonsen—305, Presidente Prudente, São Paulo CEP 19060-900, Brazil

## Abstract

Binary blends of both commercial petrodiesel and biodiesel were prepared and characterized at room temperature. Petrodiesel-type ultralow sulfur with a Sulfur level of 10 ppm/kg called S10 was used, while biodiesel was a methylic one synthesized via soybean oil and methanol, with a composition based on a mixture of several molecules-type fatty acid monoalkyl esters. A set of 10 binary blends containing 0 to 100% biodiesel were prepared by conventional mixing of volumetric fraction of components. The structural characterization of blends was carried out by attenuated total reflection Fourier transform infrared spectroscopy, ATR-FTIR, in the Mid region. Volumetric properties of the blends were measured via the pycnometry technique. Thermal conductivity was investigated via a digital thermal conductivimeter, using a two-needle sensor. Infrared spectra of all blends exhibited a significant shifting of the position of the characteristic absorption band of biodiesel, a specific vibration of the charged head assigned to esters groups. Values of parameters intensity, area, and specific absorption bands of diesel and biodiesel and volume of each blend, as well as thermal conductivity, undergo changes as a function of biodiesel fraction in the mixture. Evolution of parameters mentioned as a function of the biodiesel fraction in the blend was assigned to the molecular interaction between the molecule components of the mixture. In addition, these values do not obey a linear simple mixture rule. However, the set of structural, densimetric/volume, and thermal data of binary blends show the development of Excess phenomena properties that are associated with liquid cohesion. Based on the secondary chemical bond concept, the properties of Excess were discussed.

## Graphical abstract

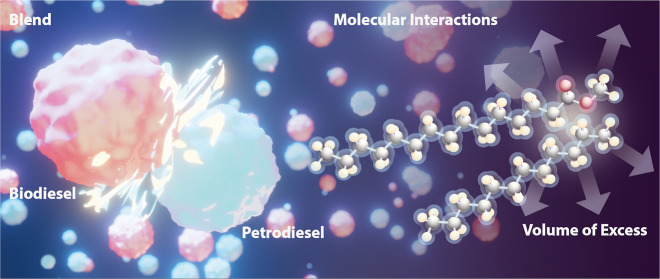

## 1 Introduction

Nowadays, a great number of investigations involving synthesis methods, catalysts, raw materials, and some properties of biodiesel have been carried out.^1–5^ Acid–base bifunctional heterogeneous solid catalysts, based on niobium and alkali metal oxides as M_*y*_NbO_*X*_ and M_2_ZrO_2_ (M = Li, Na, and K), have shown good performance on the transesterification for soybean oil for green fuel production.^2,6^ In this sense, several effective catalysts produced from soil and lithium carbonate have demonstrated efficiencies toward transesterification reactions.^7,8^ In addition, other catalysts based on Li_2_CO_3_ and rice husks as Li_4_SiO_4_ solid-base catalysts for biodiesel production have presented one of the most effective catalytic systems available for improving biodiesel by using RHA and a lithium carbonate composite.^9^

Further, a significant number of studies have been addressed to thermodynamic aspects and its effects on the mechanics of biodiesel/diesel blends,^3,10–12^ examining how thermodynamic properties^13,14^ influence engine performance^3,11^ and contribute to the optimization of engine components.^15–17^

As a matter of fact, physical-chemistry features and structural and thermal conductivity properties of blends are strategic to the understanding of properties and designing of new fuel blend-type biodiesel/petrodiesel, as well as enhancement of the blend’ performance by the selection of additives. The development of biodiesel/petrodiesel blends is a key strategy for reducing the reliance on fossil fuels and lowering emissions. Recent research focuses on optimizing blend properties, engine performance, and emissions through careful selection of feedstocks, blend ratios, and innovative additives, including nanoparticles and oxygenated compounds.^18–20^

Binary blends of type biodiesel/diesel have been strategic from a well-designed and structured energetic matrix. We take into account binary blend biodiesel/petrodiesel, a biofuel/fuel, in which both biofuel and fuel are classified as functional compounds of a long carbonic chain due to intrinsic characteristics. These mixtures have been extensively studied due to their potential to reduce emissions and improve fuel properties. Recent studies have addressed aspects ranging from physicochemical properties and molecular behavior to engine performance and environmental and health impacts.^21,22^

Biodiesel is characterized as an alkyl ester, essentially a mixture of ester compounds, with a weakly polar nature due to the small dipole moment of its charged headgroup, whereas diesel is composed of hydrocarbons, in fact a mixture of hydrocarbon molecules. The long carbon chain imparts a wide range of interesting properties to this class of molecules, enabling them to exhibit quasipolar behavior with a small charge density distributed along the linear portion of the molecule. From this point, the classical golden rule of solubility “polar compounds dissolve polar compounds and vice versa”. In this sense, petrodiesel and biodiesel are mutually soluble at all extensions of volumes. Such characteristics can be tailored to the development of an infinite number of biofuel blends based on biodiesel and applications. However, petrodiesel has composition that depends on the feeds’ source, processing, and specifications.^23^ The composition of petrodiesel and similar fuels changes in a significant way depending on the origin of the raw materials, the refining processes, and compositional adjust. This variability directly impacts physicochemical properties, engine performance, and emissions, making it essential to understand how each step influences the final product.^24,25^

In a broad sense, petrodiesel is a mixture of alkanes, aromatic/polyaromatics hydrocarbons, and cycloalkanes, typically compounds containing from 12 to 22 carbon atoms. During the desulfurization process, petrodiesel undergoes further compositional changes due to elimination of a set of molecules that exhibit sulfur in its molecules as thiols, sulfides, cyclic sulfides, disulfides, benzothiophenes, dibenzothiophene, and naphtha benzothiophene, which have a permanent dipole moment with strong influence on the liquid packing. Thus, due to the systematic removal of a significant portion of polyaromatic molecules (the main sulfur sources), diesel is classified as having a low sulfur level. For instance, this is observed during the refining of petrodiesel to obtain ultralow sulfur diesel. Lowering the sulfur content to around 10 ppm not only guarantees cleaner diesel engine emissions but also substantially decreases the fraction of molecules with dipolar properties. In this sense, the removing of sulfur leads to decreasing of lubricity due to the rendering of polycyclic aromatics that contain nitrogen and oxygen,^23^ electrical conductivity,^23^ and thermal stability. In this way, as a function of changing of composition, another set of properties can be expected when, also, in a broad sense, biodiesel exhibits a complex composition that depends on the raw materials and synthesis process. One of the most well-behaved compositions stems from the methylic route of synthesis, where a transesterification reaction is used to transform vegetable oil and alcohol methylic in biodiesel. Composition is given by a mixture of fatty acid monoalkyl esters characterized by a long chain containing 16 to 19 carbon atoms, which can be a saturated methyl palmitate that contains 16 carbon atoms and zero unsaturation (C16:0), methyl stearate (C18:0), unsaturated compounds as methyl oleate (C18:1), methyl linoleate (C18:2), and methyl linolenate (C18:3). As a minor fraction component compound, biodiesel can contain methyl arachidate (C20:0) and methyl behenate (C22:0).

In this work, a set of binary blends based on ultralow sulfur diesel (petrodiesel, a refined fossil fuel called S10, 10 ppm of sulfur) and soy-bean biodiesel synthesized via the methylic route was prepared, at which structural, thermal, and volumetric parameters were investigated, at room temperature. Properties of thermal conductivity and volume were used in an innovative way to investigate the nonlinear and anomalous properties of the mixture of blends ascribed to the Excess phenomenon. Excess properties are investigated as a function of molecular interaction. Taking into account both physics and chemistry contributions, technological and scientific aspects are discussed.

## 2 Materials and Methods

### 2.1 Materials

Both commercial, ultralow sulfur content petrodiesel ULSD called S10, 10 ppm/kg, of sulfur and biodiesel were provided by a regional fuel distributor (Small). The process of decreasing sulfur content has been carried out via hydro-desulfurization.

The commercial biodiesel was produced from methanol and soybean oil, and its water content is 112.1 ppm, according to the supplier.

Both experimental densities and thermal conductivities of these two pure components are listed in Table 1, as well as data collected from refs 3, 11, and 26–35 and the limits of the densities determined by the European Committee for Standardization (CEN)^36,37^ and the Brazilian National Agency of Petroleum, Natural Gas and Biofuels (ANP).^38,39^

**Table 1:** List of Parameters of Experimental Density (ρ_m_) and Thermal Conductivity (*k*_m_) of Biodiesel and Petrodiesel S10, at Room Temperature

	density—ρ_m_ (g cm^–3^)		limits	
	this study	range in literature	EN 14214 (CEN)	RANP 45-2014 (ANP)
biodiesel	0.881 ± 0.001	0.872–0.914 ^3,11,13,26–32^	0.860–0.900 ^37^	0.850–0.900 ^38^
			EN 590 (CEN)	RANP 50-2013 (ANP)
diesel	0.825 ± 0.001	0.811–0.850 ^3,26–31,35^	0.820–0.845 ^36^	0.815–0.850 ^39^

	thermal conductivity—*k*_m_ (W m^–1^ K^–1^)	
	this study	literature
biodiesel	0.147 ± 0.001	0.154;^33^ 0.178 ^34^
diesel	0.129 ± 0.001	

Both the petrodiesel and biodiesel infrared spectra are shown in Figure 1, while its absorption band position and its assignments are listed in Table 2.^27,40–45^ The biodiesel fatty acid composition profile is assumed to be alike an average published by Hoekman et al.^1^ and adapted to analysis listed in Table 3.

**Figure 1:**
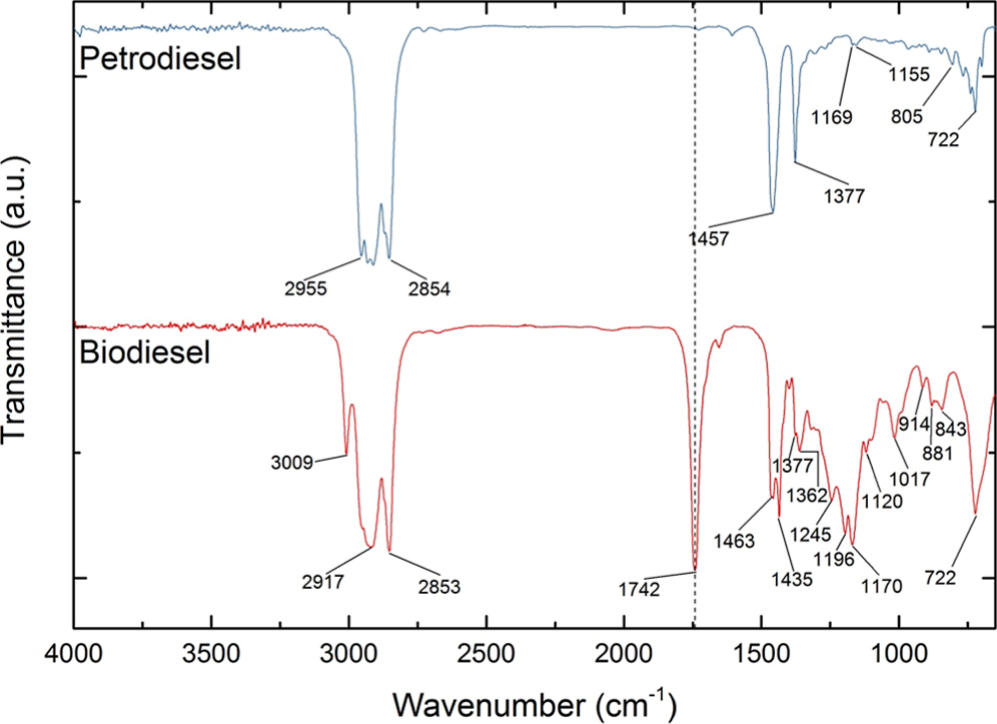
ATR-FTIR spectra of Petrodiesel and Biodiesel, at the mid region, from 4000 cm^–1^ to 650 cm^–1^.

**Table 2:** List of Positions and Its Intensities of Absorption Bands from Both ATR-FTIR Spectra of Biodiesel and Petrodiesel, as Well as Specific References^a^

		intensity		
wavenumber (cm^–1^)	vibrational mode	biodiesel	petrodiesel	references
3009	C–H str. of cis-double bond	medium	absent	^41^
2971–2853	C–H sym. and asm. str.	high	high	^27,40–42^
1742	C=O str.	high	absent	^43,45^
1457	C–H asym. bend.	absent	high	^27,41^
1377	CH_2_ sym. bend.	low	medium	^40,41^
1170	C–O str.	high	low	^41,44^
722	CH_2_ rock	medium	low	^40–42^

a Acronyms used are stirring (str), symmetric (sym), asymmetric (asm), and bending bend.

**Table 3:** List of Most Common Name, Compound Name, Carbon C: Insaturation Ratio of Molecular Species, and Its Soybean Biodiesel Average Composition

	fatty acid			
common name	compound name	C/insaturation	average (weight %)	molecular weight
linoleic	*cis*,*cis*-9,12-octadecadienoic acid	18:02	53.8	280.46
oleic	*cis*-9-octadecadienoic acid	18:01	23.7	282.47
palmitic	hexadecanoic acid	16:00	11.6	256.43
linolenic	*cis*,*cis*,*cis*-9,12,15-octadecatrienoic acid	18:03	5.9	278.44
stearic	octadecadienoic acid	18:00	3.9	284.48
arachidic	icosanoic acid	20:00	0.3	312.54
gondoic	*cis*-11-eicosenoic acid	20:01	0.3	310.53
behenic	docosanoic acid	22:00	0.3	340.6
nervonic	*cis*-15-tetracosenoic acid	24:01	0.3	366.62
palmitoleic	*cis*-9-hexadecenoic acid	16:01	0.2	254.42
lauric	dodecanoic acid	12:00	0.1	200.32
myristic	tetradecanoic acid	14:00	0.1	228.38
heptadecanoic	heptadecanoic acid	17:00	0.1	268.43
erucic	*cis*-13-docosenoic	22:01	0.1	338.58
lignoceric	tetracosanoic	24:00	0.1	368.64
	other/unknown		4.1	

### 2.2 Methods and Experimental Procedure

A micropipette was used for volume measurements. The set of homogeneous blends were prepared from mixtures between 0 and 100% of biodiesel with steps of 10% in volume and mixed in a vortex mixer. A set of 11 samples of blends were prepared. An investigation secondary shows that after a storage time of 2 days, no phase separation was observed at room temperature, as expected.

Densities of samples were determined using a pycnometer with a volume of 5 cm^3^ and a thermometer and an analytical balance SHIMADZU ATY224 that has an uncertainty of 0.2 mg; the formation and cumulating of air bubbles were avoided. The volume of the pycnometer was taken from calibration with distilled water, and densities were calculated following eq 1:

1 $$V=\left(W_{f}-W_{e}\right)\frac{1}{\rho_{w}-\rho_{a}}\left(1-\frac{\rho_{a}}{\rho_{c}}\right)\left[1-\gamma \left(T_{w}-T\right)\right]$$

where *V* is the volume of the pycnometer at temperature *T* (°C), *W*_f_ and *W*_e_ (g) are the masses of the pycnometer full of water and empty, respectively, and ρ_w_, ρ_a_, and ρ_c_ (g/cm^3^) are the water densities, air, and calibration weight, respectively. γ (1/°C) represents the volumetric coefficient of thermal expansion of the material from which the pycnometer is made, and *T*_w_ represents the temperature of water, in the calibration.

2 $$\rho_{w}=a_{5}\left[1-\frac{{\left(T+a_{1}\right)}^{2}\left(T+a_{2}\right)}{a_{3}\left(T+a_{4}\right)}\right]$$

where *a*_1_, *a*_2_, *a*_3_, *a*_4_, and *a*_5_ ^46^ are constants given in Table 4.

3 $$\rho_{a}=\frac{C_{1}p_{a}+h_{r}\left(C_{2}T+C_{3}\right)}{T+273.15}$$

where ρ_a_ (g cm^–3^) represents the density of air at *T* (°C), *h*_r_ represents the relative humidity (%), *p*_a_ (hPa) represents the atmospheric pressure, and *C*_1_, *C*_2_, and *C*_3_ ^33^ are constants also listed in Table 4.

4 $$\rho_{m}=\frac{W_{l}}{V\left[1+\gamma \left(T_{m}-T\right)\right]}\left(1-\frac{\rho_{a}}{\rho_{c}}\right)+\rho_{a}$$

where ρ_m_ (g cm^–3^) is the density of a liquid mixture at *T*_m_ (°C), *V* is the volume of the pycnometer at *T* (°C), *W*_l_ is the mass of the liquid contained in the pycnometer, ρ_a_ and ρ_c_ (g cm^–3^) are the densities of air and calibration weight, respectively, and γ (1/°C) denotes the volumetric coefficient of thermal expansion, as described elsewhere.

**Table 4:** List the of Set of Values of Constants *a*_1_, *a*_2_, *a*_3_, *a*_4_, *a*_5_, *C*_1_, *C*_2_, and *C*_3_ Obtained from eqs 2 and 3

constant	value	references
*a*_1_ (°C)	–3.983035	^46^
*a*_2_ (°C)	301.797	^46^
*a*_3_ (°C)^2^	522528.9	^46^
*a*_4_ (°C)	69.34881	^46^
*a*_5_ (g cm^–3^)	0.999974950	^46^
*C*_1_ (°C hPa^–1^)	3.4844 × 10^–4^	^47^
*C*_2_ (g cm^–3^)	–2.52 × 10^–6^	^47^
*C*_3_ (°C)	2.0582 × 10^–5^	^47^

The uncertainty of density measurements is estimated to be equal to ±0.001 g cm^–3^. In this work, all measurements were made at a temperature of 298.15 K, atmospheric pressure, and in a triplicate way. The average valor of the measurements is exhibited.

### 2.3 Thermal Conductivity Characterization

Thermal conductivity *K* (W mK^–1^) values of biodiesel, petrodiesel, and all blends were measured using the KD2 Pro Thermal Properties Analyzer, which uses the Transient Hot-Wire THW technique. This equipment complies with the international standards EN62326:2013 and EN50581:2012, according to both operator’s manual version February 29, 2016, and ASTM D5334, as well as IEEE 442-1981.^48^ A good precision using KD2 Pro at 298.15 K has been reported.^49,50^ For deriving the thermal conductivity property was used eq 5:

5 $$K=\frac{q}{4\pi m}$$

where *K* (W mK^–1^) represents the thermal conductivity, *q* (W) is the amount of heat produced per unit of time, and *m* is the slope coefficient of the straight line. Since heat transfer in liquids can occur through conduction and convection, in the KD2 Pro, at 298.15 K,^49,50^ both convective fraction needs to be as minimized as possible.^48^ In accordance with the description in the equipment’s operator’s manual guidelines, the free convection in KD2 Pro is minimized. The temperature of each sample was stabilized with a thermostatic bath fully loaded with deionized water (about 10 L) and had the heating and pump system turned off seconds before that the measurement was taken place. Before every measure, the temperature stability of the sample was monitored after stabilization for 30 s and its variation should not be higher than 0.01 K. Thermal conductivity measurements were carried out for the each one of the fuels for comparative purposes, and its uncertainty is estimated to be <0.001 W mK^–1^.

### 2.4 Structural Characterization of Liquids via Chemical Bonds

Chemical bonds were investigated by attenuated total reflectance Fourier transform infrared spectroscopy (ATR-FTIR). It is a versatile and simple-to-use technique with many applications reported in the literature.^51–55^ Spectra were corrected for dark current noises and background using smoothing and multipoint baseline corrections with Shimadzu’s software IRsolution. Each sample was placed on a trough crystal plate, consisting of a zinc selenide prism with a 45° angle, providing 10 internal reflections. Infrared spectrum was collected with a resolution of 2.0 cm^–1^, Happ-Genzel function for apodization, and averaging over 120 scans using a Fourier transform spectrometer model SHIMADZU IR Affinity-1, in the range of 4000–650 cm^–1^.

#### 2.4.1 Spectrum Correction

For spectrum correction of the Abnormal Band Intensity, a common phenomenon in ATR measurements, a normalization technique toward a single selected wavelength was applied. It is a scatter-corrective method, which means that the variability between samples due to scatter is reduced^56^ and therefore bands can be better compared. The wavelength of 2263.3 cm^–1^ was used as a reference to 100% of transmittance, and then the spectrum was recalculated point-by-point using the correlation given in eq 6

6 $$T_{n}=\frac{T_{\nu }}{T_{\nu ,ref}}\times 100\%$$

where *T*_*n*_ represents the transmittance and *n* means normalized in the wavenumber **ν**.

#### 2.4.2 Theoretical Spectra of Blends

A theoretical transmittance spectrum of each blend was derived by the Simple Mixture Rule (SMR), using the transmittance data, point-to-point, collected from each components of the blend, in this work biodiesel and diesel S10, see Figure 1, which is a simplified prediction of mixture properties analogous to a classical Pawlewski’s rule (*T*_SMR_)^43^

7 $$T_{SMR}=\sum_{i=1}^{2}\phi_{i}T_{i}$$

where *T*_SMR_ is the blend transmittance, which was calculated classical transmittance and ϕ is the volumetric fraction of biodiesel ϕ_Β_ and diesel S10 being the summation of fractions ϕ_Diesel_ + ϕ_Β_ = 100. Then, a blend with 80% vol of diesel ϕ_Diesel_ has 20% in vol of biodiesel ϕ_Β_ have its *T* derived as follows:

7a $$T_{blend80:20}=\phi_{\left\{\{Diesel\}\right\}}T_{diesel}+\phi_{\left\{\{Β\}\right\}}T_{biodiesel}={0.8T}_{diesel}+0.2T_{biodiesel}$$

As a matter of fact, only a part of the transmittance spectrum has been used. Here, only a region between 1880 and 1700 cm^–1^ close to biodiesel fingerprint positioned at wavenumber 1472 cm^–1^, as shown in Figure 1. Set of equations similar to eq 7a, with ϕ_Β_ = 10, 20, 30, 40, 50, 60, 70, 80, and 90% in vol was used to development of blends investigated, see Figure 3b,d.^57^

Normalized spectra, experimental and theoretical, were represented in the absorbance spectrum in order to use the software Peak-Fit for the quantitative analysis by the following formula:

8 A=2−log⁡T

where *A* represents the absorbance and *T* represents the transmittance.

#### 2.4.3 Spectra Analysis via Band Deconvolution

A quantitative analysis of infrared spectra was carried out by a careful deconvolution of the absorption profiles using the software Origin and Peak-Fit that considers hidden peaks at wavenumbers different from the local maximum in the data stream.^58^ As a matter of fact, this feature does not mean that a hidden peak is not discernible.

For the sake of completeness, the deconvolution procedure was applied to a specific interval of the spectrum range. This range is positioned in the part of the mid-infrared, where both characteristic absorption bands assigned to biodiesel and petrodiesel occur.

For the procedure of data adjustment was used the Lorentzian distribution to each absorption band. In one dimension, the Lorentz probability density function is given by the relation:

9 $$f\left(x;x_{c},\gamma \right)=\frac{1}{\pi \gamma \left[1+{\left(\frac{x-x_{c}}{\gamma }\right)}^{2}\right]}$$

where *x* represents the independent variable, *x*_c_ is the band’s center, and 2γ is the full width at half-maximum fwhm parameter. Equation 9 must be rewritten to enable variable values for the band’s area *A*:

10 $$f\left(x;x_{c},\gamma ,A\right)=\frac{A}{\pi \gamma \left[1+{\left(\frac{x-x_{c}}{\gamma }\right)}^{2}\right]}$$

In order to explicit fwhm (2γ), the function becomes

11 $$f\left(x;x_{c},\gamma ,A\right)=\frac{2A}{\pi }\left[\frac{2\gamma }{4{\left(x-x_{c}\right)}^{2}+{\left(2\gamma \right)}^{2}}\right]$$

The amplitude *I*_*c*_ is obtained using eq 12:

12 $$I_{c}=\frac{A}{\pi \gamma }$$

The apparent absence of hidden bands was verified by using Peak-Fit by a residual tool. Amplitude (*I*_c_), area (*A*), position (*x*_c_), and fwhm (2γ) of the band of interest were derived by theoretical adjustment using eq 10, in the Origin software. Area *A* values plotted were relative to the area of the characteristic absorption bands of B100, biodiesel. The same was considered true for the amplitude *I*_c_ values. These parameters are shown in Figure 2 for better visualization. As a whole, all absorption bands exhibit a very small distortion, being that the observed profile is very close to the Gaussian function with high symmetry. The adjustment routine is based on minimization of deviations between the experimental and simulated spectra. Data on the band positions detected automatically by the spectrometer were used as an input file. A linear baseline was subtracted from all spectra.

**Figure 2:**
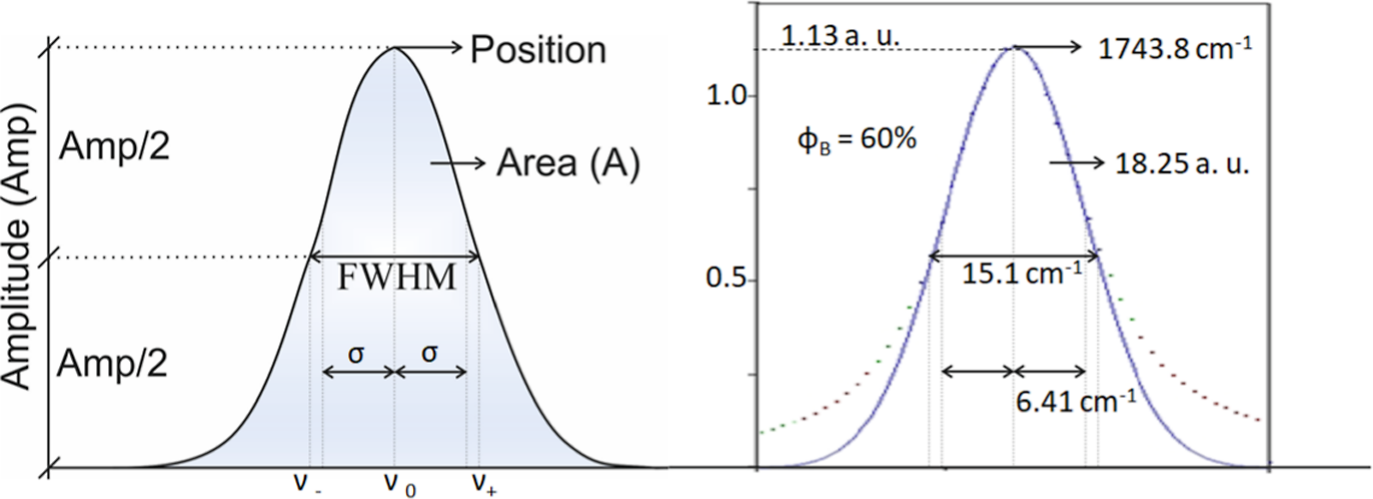
Scheme for visual identification of parameters: on the left side, Amp denotes the amplitude, *A* the integrated area, position the band center, and fwhm the full width at half-maximum of an absorption band; high side parameters Amp = 1.13 au, area = *A* = 18.25 au, and fwhm = 15.1 cm^–1^, values derived for blend with blend diesel 40% and biodiesel 60% in volume, **ϕ**_Β_.

## 3 Results and Discussion

### 3.1 ATR-FTIR Spectroscopy of Biodiesel/Petrodiesel Blends

The structural investigation of blends was carried out by ATR-FTIR. Physical parameters of the characteristic bond of biodiesel, assigned of the esters group, were used as a molecular interaction probe.^27,59^ In Infrared spectroscopy, the wavenumber (ν̅) represents the reciprocal of the wavelength λ:

13 $$\mathrm{\nu ̅}=\frac{1}{\lambda }$$

where ν̅ is in units of cm^–1^ since λ is in units of cm. Both parameters wavelength λ and frequency, *f*, can be related by eq 14:

14 $$f=\frac{c}{\lambda }$$

where the parameter *c* represents the speed of light, which is at about 3.00 × 10^10^ cm/s. Further relation between parameters *f* and λ is found by the substitution of eq 13 into eq 14, as follows:

15 f=ν̅c

According to eqs 13 and 14, short wavelength λ represents the more energetic position in the electromagnetic spectrum and vice versa. At the interval of analysis from 4000 cm^–1^ and 650 cm^–1^, the shifting of a band position to the minor wavenumber side means that the absorption band shifts to the low energetic side of the spectrum, whereas a shift toward higher wavenumber means that one shifts to the more energetic side of the spectrum. The wavenumber can be correlated with energy per photon *E* by Planck’s relation:

16 E=hf=hcν̅

where *h* represents Planck’s constant and *E* is directly proportional to the wavenumber.

Figure 3 shows several sets of ATR-FTIR spectra of mixtures at 9 blends, as a function of biodiesel volumetric fraction ϕ_B_, of both experimental and theoretical FTIR spectra. Figure 3a and Figure 3c show the experimental spectra of mixtures in two spectral regions. The first region is positioned between 1800 and 1700 cm^–1^, such a region contains the fingerprint band of biodiesel, while the second region is positioned between 3050 and 2980 cm^–1^. In addition, theoretical ATR-FTIR spectra of Figure 3b,d are shown at the same intervals of wavenumber with each spectrum derived from the classical mixture rule, as described in item Section 2.4.2.

**Figure 3:**
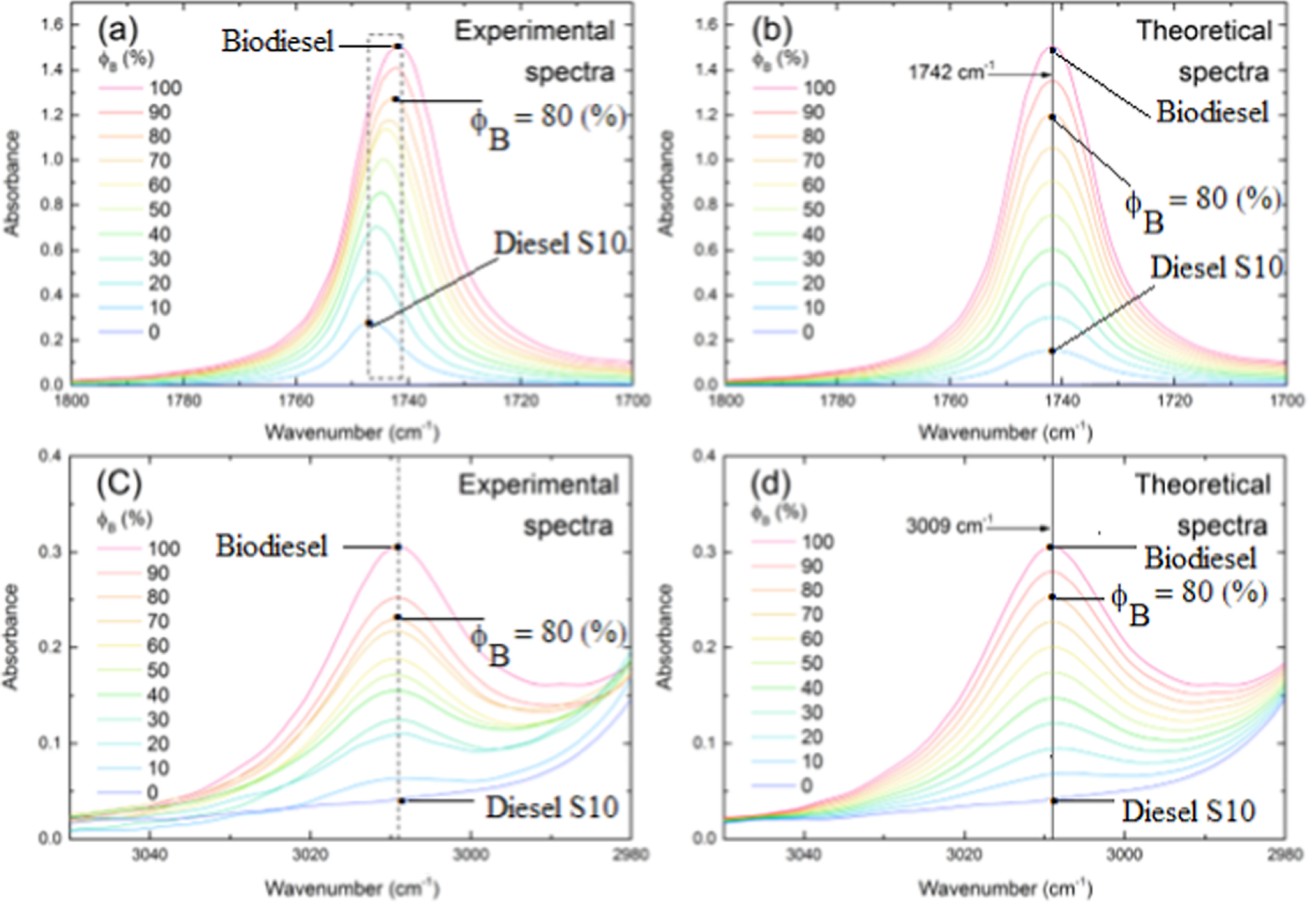
ATR-FTIR spectra of biodiesel (ϕ_Β_ = 100%)/petrodiesel (ϕ_Β_ = 0) and its blends ϕ_Β_ = 10, 20, 30, 40, 50, 60, 70, 80, and 90% in vol, at around the region of position of biodiesel fingerprint (1742 cm^–1^), 1800 cm^–1^ to 1700 cm^–1^, (a) experimental spectra and (b) theoretical spectra. Portion of spectra shown in wavenumber between 3050 and 2980 cm^–1^ and (c) experimental and (d) theoretical spectra were derived using the SMR, see item Section 2.4.2.

Figure 3a shows that a complex absorption band evolution is apparent from the first blend composition (ϕ_B_ = 10%). Each spectrum exhibits a shift in band position from 1747 cm^–1^ to 1742 cm^–1^, a phenomenon highlighted in the figure with a dashed window.

As discussed in Figure 1, the band centered at 1742 cm^–1^ is a characteristic band or fingerprint of the biodiesel, assigned to the C=O stretching of the ester group. According to Figure 3a, this band shows a nonlinear intensity, proportional to the fraction of biodiesel in the mixture, evolution indicating the existence of further contribution to the spectrum distortion, in addition to the mass amount of biodiesel in the mixture, see ϕ_Β_. Furthermore, in addition to the band shift, a complex evolution of the band position is observed as a function of the biodiesel content in the mixture.

The band area also exhibits a complex evolution. The simultaneous changes in band position, intensity, are further detected in the band area, providing clear evidence of molecular interaction phenomena.

Figure 3b shows spectra stemming from the classical mixture rule that provides a collection of spectra with an “ideal” behavior, in which molecular interaction does not exist, only the mass action law. Then, as expected, the band position assigned to the ester group is invariant. Moreover, both the intensity and area evolution as a function of biodiesel fraction are linear. Figure 3c shows also a set of experimental ATR-FTIR spectra assigned to a noncharged portion from the molecule, an inner vibrational band belonging to a linear chain of ester, C–H stretching, which is centered at 3009 cm^–1^ at all blends composition. Also, according to Figure 1, the band centered at 3009 cm^–1^ is identified as a secondary characteristic band of biodiesel. As expected, this band shows a particular intensity evolution ascribed to the mass amount of biodiesel in the mixture and only a very slight effect associated with the area. Any shifting of the band occurs. Spectra derived from the classical mixture rule involving the band centered at 3009 cm^–1^ are shown in Figure 3d. The intensity and area of the vibrational band are well behaved, indicating that molecular interaction does not exist when theoretical spectra are further considered.

Figure 4 shows both experimental and theoretical spectra for selected blends at regions between 1800 and 1700 cm^–1^, grouped by the volumetric fraction of biodiesel for a further comparison. Figure 4a presents the blend containing 10% biodiesel, which exhibits the largest band shift among the blends. As there is less biodiesel in the sample, the band shifts. As a whole, experimental mixtures exhibit a distinct feature of a characteristic band involving area, position, and band intensity and then evidence molecular interactions, a phenomenon nonobservable in the theoretical spectrum.

**Figure 4:**
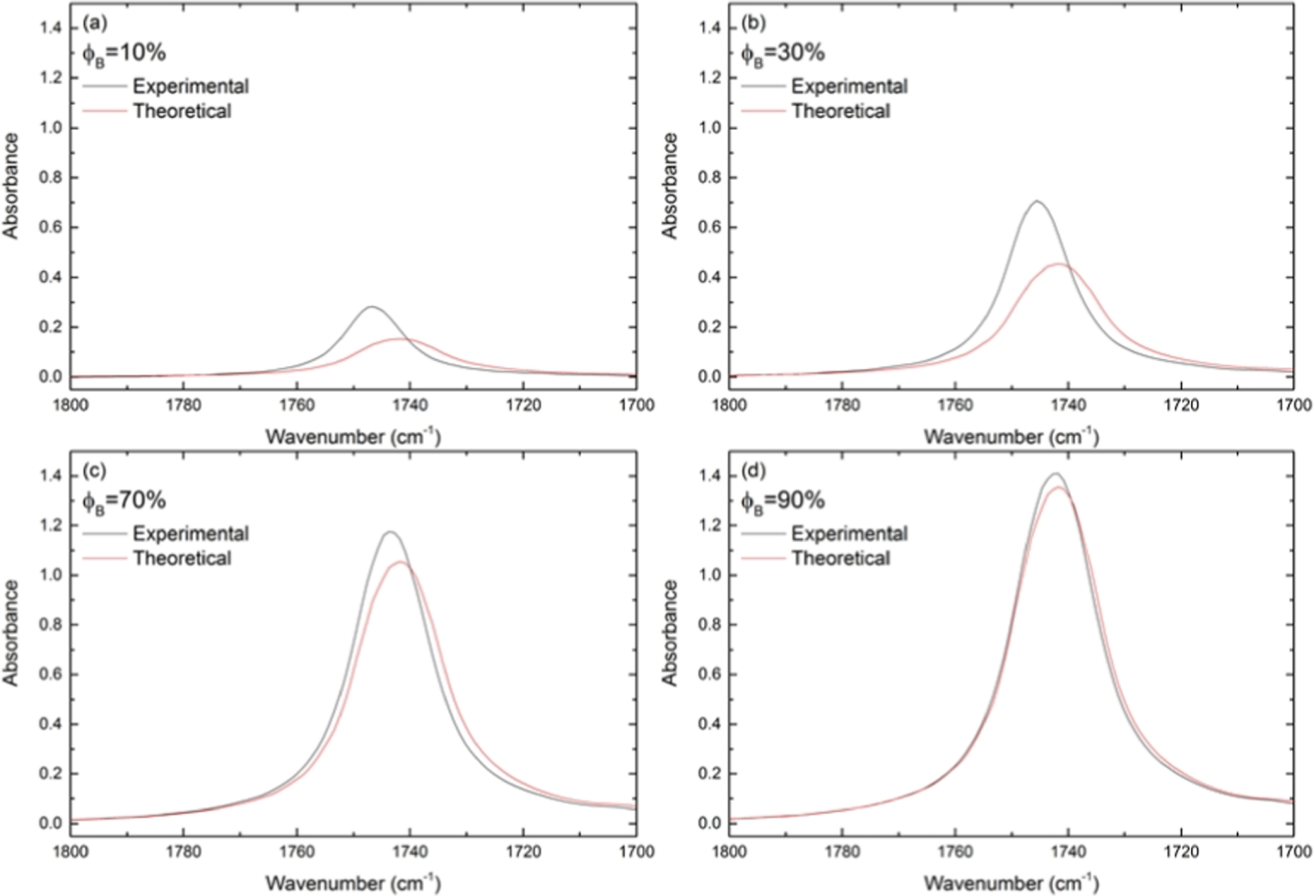
ATR-FTIR experimental and theoretical spectra of biodiesel/petrodiesel selected blends (a) ϕ_B_ = 10%, (b) ϕ_B_ = 30%, (c) ϕ_B_ = 70%, and (d) ϕ_B_ = 90% from 1800 cm^–1^ to 1700 cm^–1^. The experimental data are the black lines, and the theoretical data are plotted in red lines, derived from the SMR.

Figure 5 shows a set of examples of spectrum deconvolutions carried out in the region of existence of the absorption characteristic band assigned charged head of the ester group between 1850 and 1650 cm^–1^.

**Figure 5:**
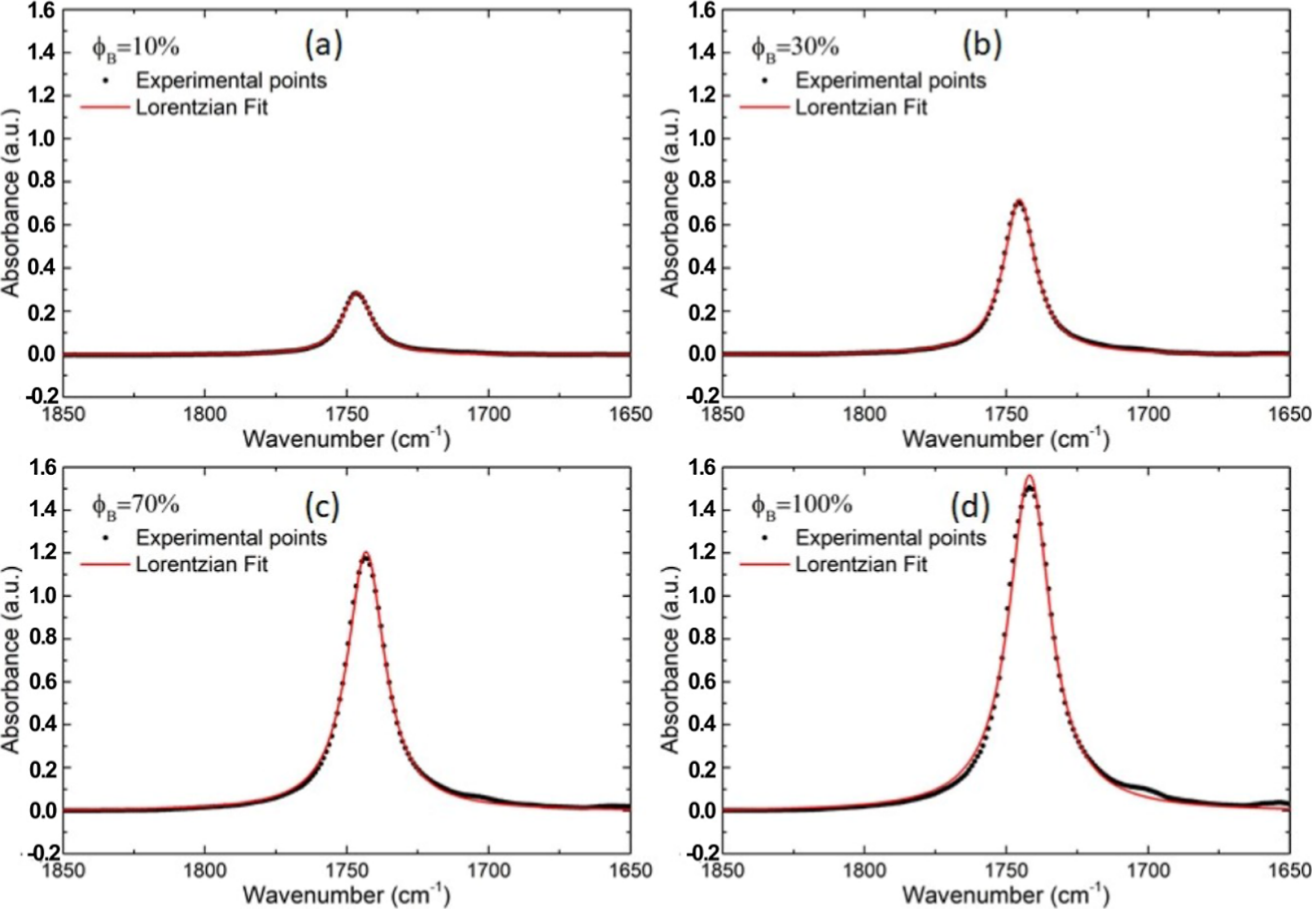
Deconvolution of ATR-FTIR absorbance spectra of biodiesel/petrodiesel selected blends (a) ϕ_B_ = 10%, (b) ϕ_B_ = 30%, (c) ϕ_B_ = 70%, and (d) ϕ_B_ = 100% in the region between 1850 and 1650 cm^–1^. The experimental data is represented by the black dots, and the theoretical adjust appears as the red line.

Both the amplitude and area parameters obtained from the spectrum deconvolutions are listed in Table 5 as the theoretical values derived from the SMR; see item Section 2.4.2.

**Table 5:** List of Experimental and Theoretical Values of Amplitude and Area for Biodiesel/Diesel Blends as a Function of Biodiesel Volumetric Fraction ϕ_B_ ^a^

	amplitude		area	
ϕ_B_ (%)	experimental	theoretical	experimental	theoretical
10	0.28	0.15	3.91	2.90
20	0.50	0.30	6.97	5.80
30	0.70	0.45	10.23	8.70
40	0.85	0.60	12.98	11.60
50	1.00	0.75	15.74	14.50
60	1.13	0.89	18.25	17.40
70	1.18	1.04	20.60	20.30
80	1.28	1.19	23.22	23.20
90	1.50	1.34	29.39	26.10
100	1.49	1.49	29.00	29.00

a Collected and derived values from the band centered at around 1742 cm^–1^ in biodiesel/petrodiesel blends.

According to Table 5, point-to-point experimental data exhibit values higher than those of the theoretical values. The evolution of both parameters’ relative amplitude and relative area derived of the characteristic band centered at around 1742 cm^–1^ of biodiesel/petrodiesel blends versus biodiesel volumetric fraction ϕ_B_ is shown in Figure 6.

**Figure 6:**
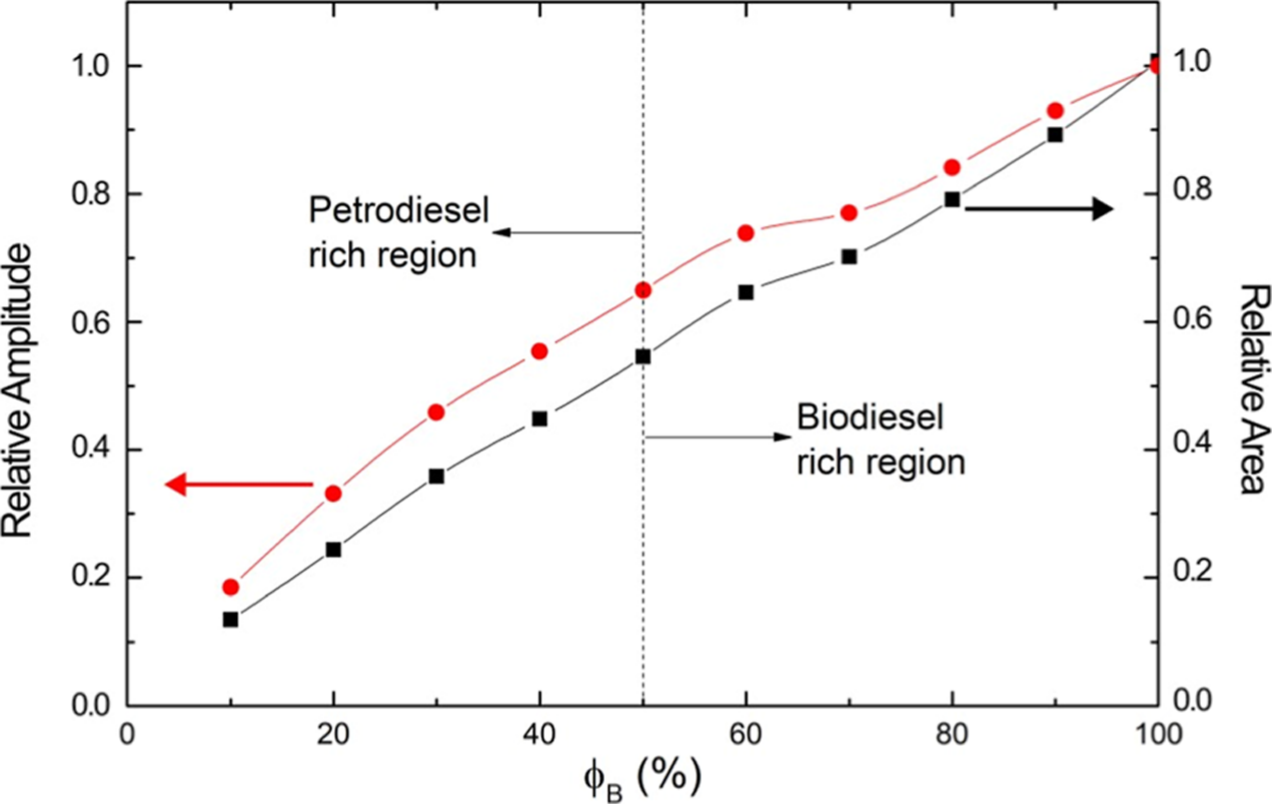
Plot of both parameters relative amplitude (dimensionless) and relative area (dimensionless) of the characteristic band centered at around 1742 cm^–1^ of biodiesel/petrodiesel blends versus biodiesel volumetric fraction ϕ_B_. Both plots are relative to the band of biodiesel. All points stemming from the experimental data.

Despite the intensity parameter being relevant, the intensity integrated or area is a reach of physical meaning. The plot of both curves is relative to the characteristic band of biodiesel (ϕ_B_ = 100%) being that a non-linear evolution of amplitude and the integrated area is easily identified. The plots of both relative amplitude and area of the characteristic band centered at around 1742 cm^–1^ of biodiesel/petrodiesel blends versus biodiesel volumetric fraction ϕ_B_ are shown in a double logarithmic scale in Figure 7. The nonlinear character of both relative amplitude (intensity) and area parameters stays observable. This behavior provides further evidence that both intensity and integrated area contain the influence of molecular interaction transporting the intrinsic properties of the blend.

**Figure 7:**
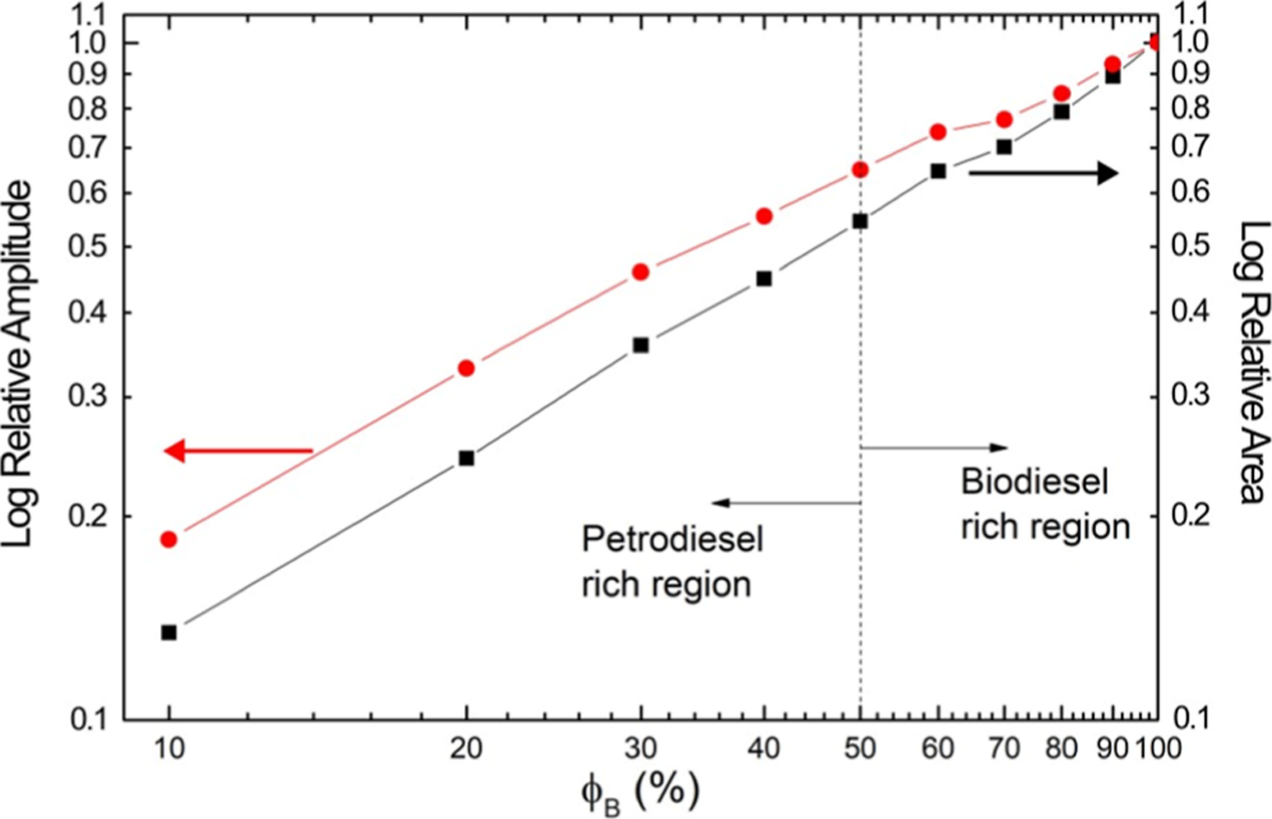
Plot in a double logarithmic scale of both relative amplitude and integrated area of the characteristic band centered at around 1742 cm^–1^ of biodiesel/petrodiesel blends vs biodiesel volumetric fraction ϕ_B_. Both curves are relative to the band of biodiesel. All points stemming from the experimental data.

The evolution of both parameters of the absorption band center and energy of the absorption band at around 1742 cm^–1^ in biodiesel/petrodiesel blends versus biodiesel volumetric fraction ϕ_B_ is shown in Figure 8.

**Figure 8:**
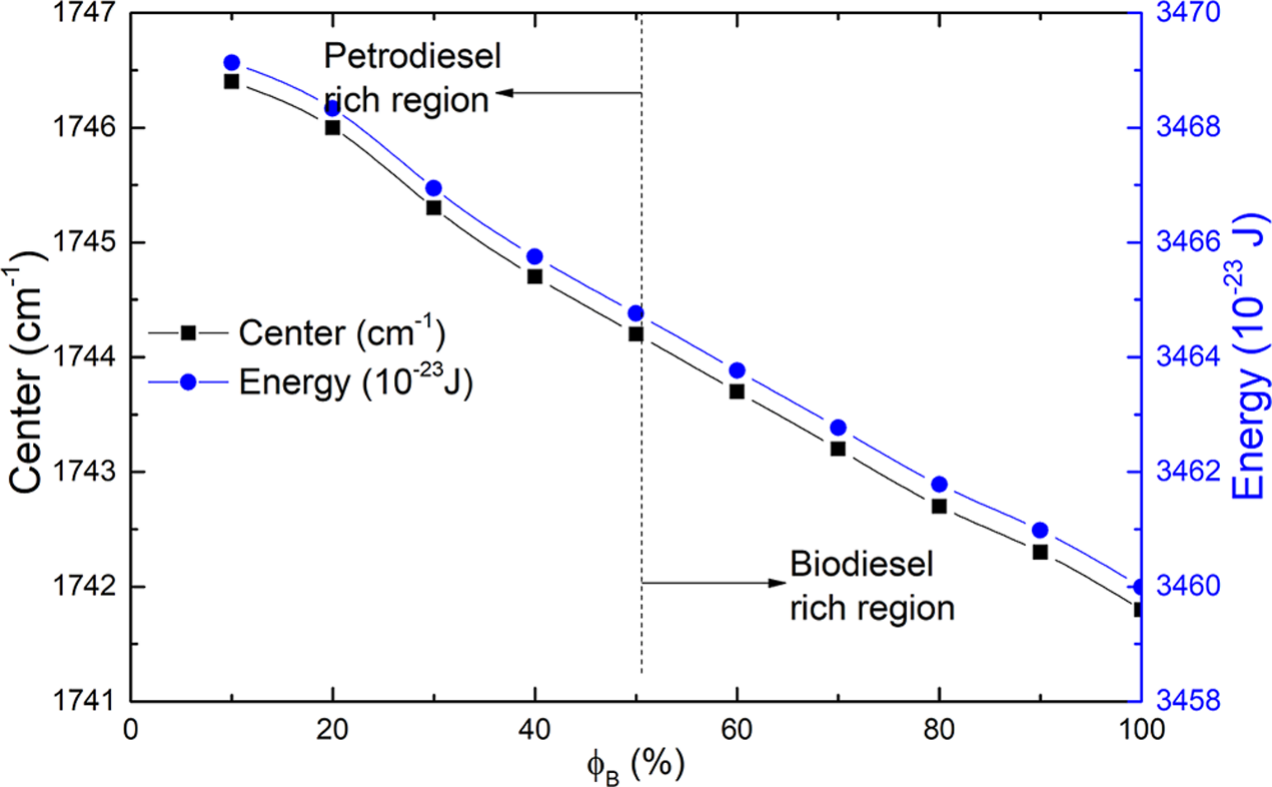
Plot of absorption band center (solid black square) and energy of the absorption band (solid blue circle) at around 1742 cm^–1^ in biodiesel/petrodiesel blends vs biodiesel volumetric fraction ϕ_B_. All points stemming from the experimental data.

The evolution parameter full width at half-maximum (fwhm) of the characteristic band centered at around 1742 cm^–1^ in biodiesel/petrodiesel blends versus biodiesel volumetric fraction ϕ_B_ is shown in Figure 9. The parameter fwhm increases in a nonlinear way up to reach values of the biodiesel. In liquids, changes in fwhm can be assigned to the strength of intermolecular interactions. Then, the number and type of molecules that compose the environment of the band change as a function of ϕ_B_ changing the parameter fwhm.

**Figure 9:**
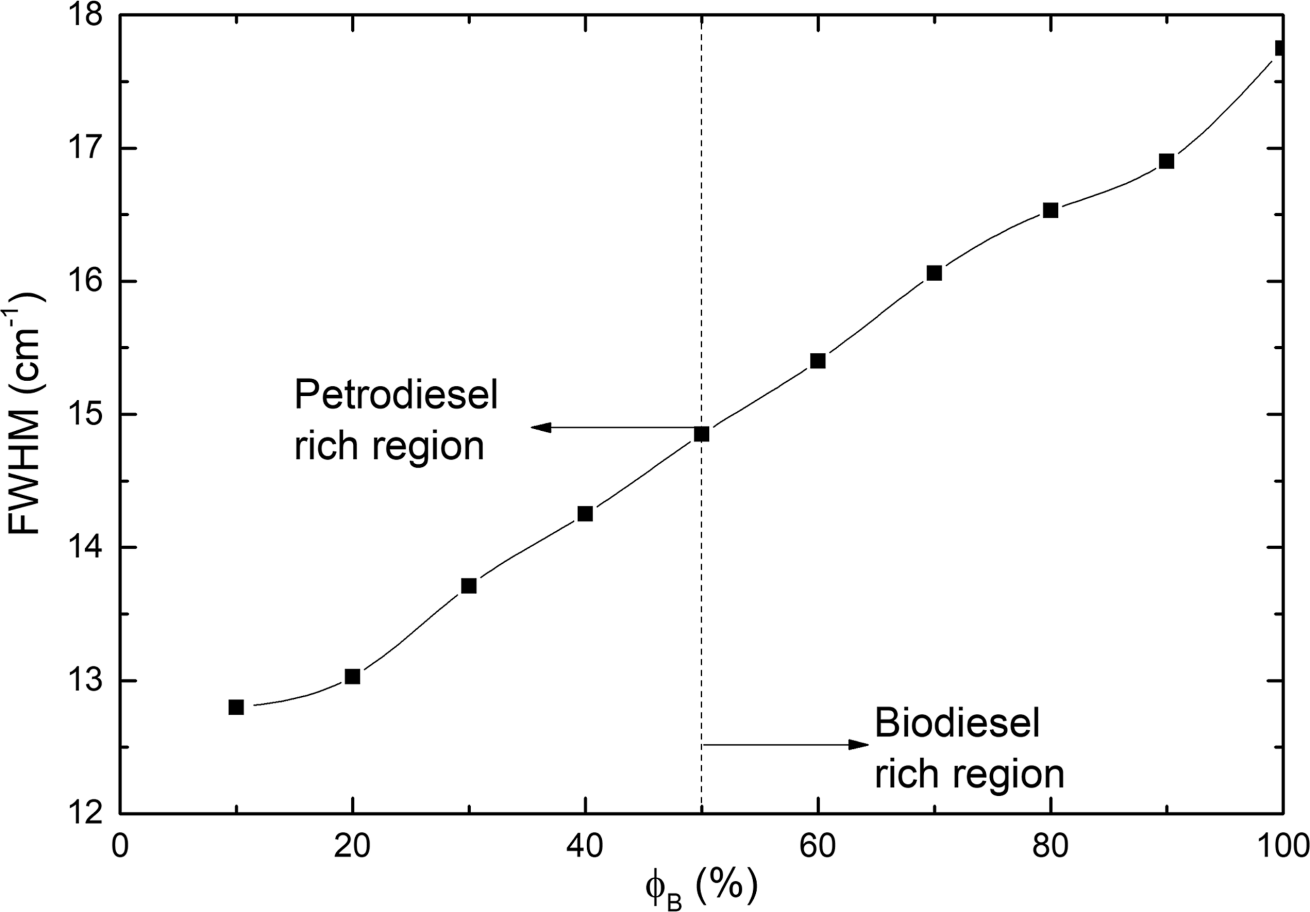
Plot of fwhm of the characteristic band centered at around 1742 cm^–1^ of biodiesel/petrodiesel blends vs biodiesel volumetric fraction ϕ_B_. Experimental data.

According to the discussions of Figures 6–9, the dependence of parameters amplitude, area, center, and fwhm of the band that started in 1742 cm^–1^ with the volumetric fraction of biodiesel in samples is in essence nonlinear, a result expected due to molecular interaction existence. As a matter of fact, none of those parameters have a true linear correlation with the volumetric fraction of biodiesel like similar reports.^27,43^ Increasing the amount of biodiesel in blends the band’s center shifts to lower wavenumbers, i.e., shifting to lower energy levels. In fact, both parameter’s absorption band position and fwhm have undergone changes as a function of ϕ_B_, which put in evidence the existence of molecular interactions, giving the natural development of Excess Properties Phenomena, in the practice is expected excess volume (expansion or retraction of blend volume), density, and thermal conductivity.

### 3.2 Density and Volume Properties

Experimental density values (ρ_m_) as a function of the volumetric fraction of biodiesel in the blends of biodiesel and petrodiesel of low sulfur content are listed in Table 6. The densities of each mixture component were used to calculate the densities of the blends by the SMR:

17 $$\rho_{SMR}=\sum_{i=1}^{2}\phi_{i}\rho_{i}$$

where ρ_r_ = ρ_SMR_ is the classical density and ϕ is the volumetric fraction of each component in the blend.

**Table 6:** Both Experimental Density (ρ_m_) and Thermal Conductivity (*k*_m_) Values for Biodiesel/Diesel Blends, as a Function of Biodiesel Volumetric Fraction ϕ_B_ ^a^

ϕ_B_ (%)	ρ_m_ (g cm^–3^)	*k*_m_ (W mK^–1^)
0	0.825 ± 0.001	0.129 ± 0.001
10	0.830 ± 0.001	0.126 ± 0.001
20	0.835 ± 0.001	0.130 ± 0.001
30	0.841 ± 0.001	0.131 ± 0.001
40	0.846 ± 0.001	0.132 ± 0.001
50	0.850 ± 0.001	0.136 ± 0.001
60	0.855 ± 0.001	0.139 ± 0.001
70	0.860 ± 0.001	0.140 ± 0.001
80	0.865 ± 0.002	0.140 ± 0.001
90	0.874 ± 0.002	0.142 ± 0.001
100	0.881 ± 0.001	0.147 ± 0.001

a Values were derived from the volumetric fraction of biodiesel.

Subscript r represents the parameter derived by the SMR and *i* represents the components biodiesel and petrodiesel of low sulfur content, *i* = 1 and 2, respectively.

As expected, see the discussion in Section 3.1, in accordance with the data listed in Table 6, experimental density values ρ_m_ differ from ρ_r_ = ρ_SMR_; in fact, ρ_m_ < ρ_r_, meaning that blends exhibit the excess density phenomenon ρ^E^. In this sense, the evolution of the density of blends can be further correlated to the volume. As an example, ρ_m_ is proportional to the reciprocal of *V*_m_, see eqs 22 and 24. The parameter *V*_m_ is derived according to eq 18:

18 $$\rho_{m}∼\frac{1}{V_{m}}$$

As a whole, the concept of density and its correlation with the reciprocal of volume allows structuring some analysis of the relation ρ_m_ – ρ_SMR_ = Excess density ρ^E^:

i. ρ^E^ > 0 ⇔ ρ_m_ > ρ_SMR_ since 1/*V*_m_ > 1/*V*_SMR_. This relation means that volume shrinkage is operational.
ii. ρ^E^ < 0, ⇔ ρ_m_ < ρ_SMR_ (ρ_r_) since 1/*V*_m_ < 1/*V*_SMR_. This relation means that a volume expansion is operational.
iii. ρ^E^ = null, implying that *V* ^E^ is null being 1/*V*_m_ = 1/*V*_SMR_. The density of the mixture is exactly the pondered summation of the isolated components’ volumes.

Plots of experimental density ρ_m_, derived as described elsewhere, and linear density, derived by the SMR and Excess molar volume *V* ^E^ of biodiesel/petrodiesel blends as a function of biodiesel volumetric fraction ϕ_B_, are shown in Figure 10. In Figure 10a–c, the experimental data are represented by red dots, and the red line represents a cubic spline interpolation of the experimental data to help a visual inspection of the evolution values. Also, in Figure 10a–c, the black line represents expected values derived from the SMR of parameters density ρ_r_ and molar volume *V*_r_.

**Figure 10:**
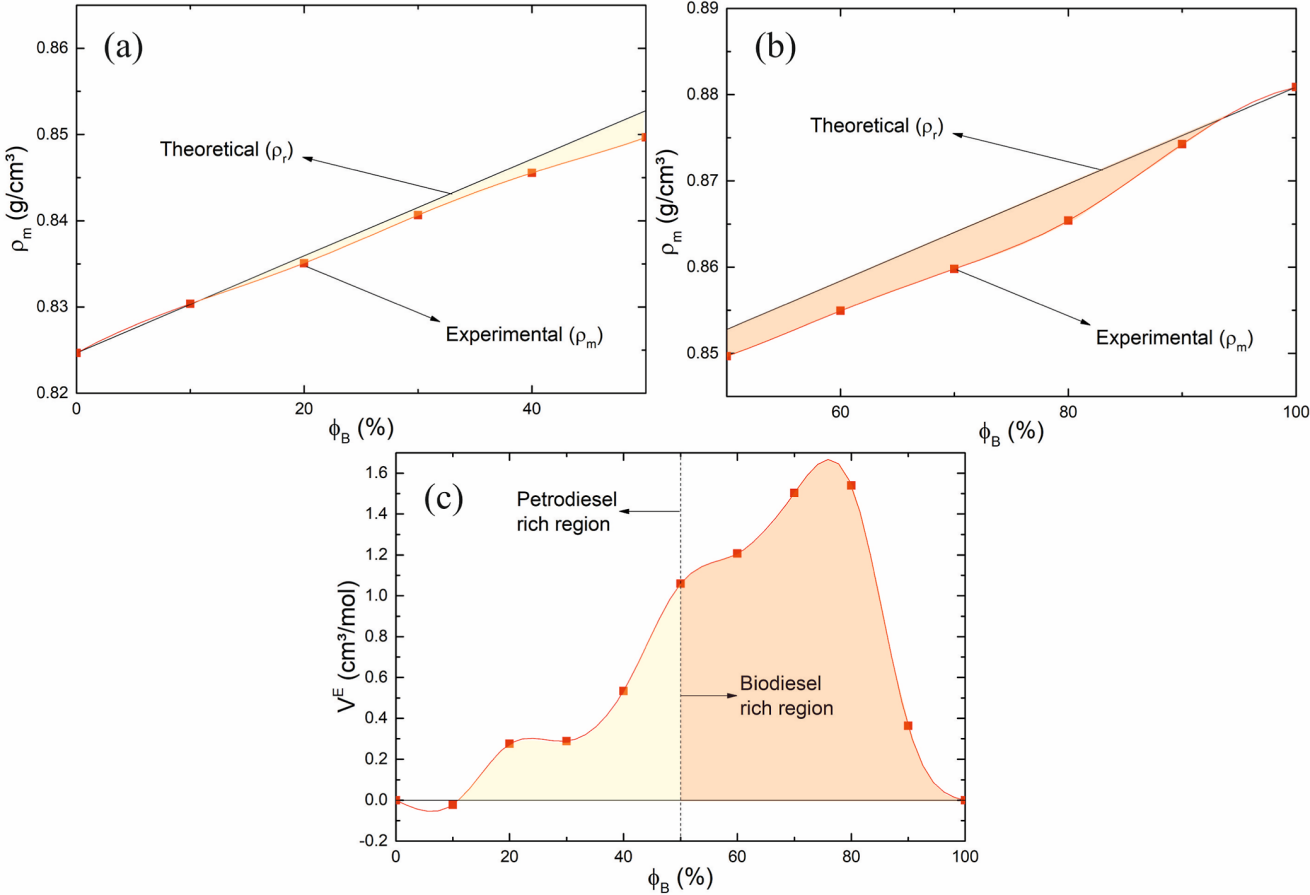
Plots of density measured ρ_m_ of biodiesel/petrodiesel blends from (a) ϕ_B_ = 0 to ϕ_B_ = 50 and (b) ϕ_B_ = 50 to ϕ_B_ = 100. (c) Plots of excess molar volume *V* ^E^ of biodiesel/petrodiesel blends vs biodiesel volumetric fraction ϕ_B_. The red dots represent the experimental data. The red line represents a cubic spline interpolation of the experimental data, and the black line represents values calculated with SMR ρ_r_ in (a,b) and zero in (c).

According to Figure 10a,b, the data show that case (ii) described above is sufficient to model the experimental data evolution. The experimental data of density ρ_m_ and its expected density valor derived via SMR ρ_SMR_ = ρ_r_ do not match. Point by point, the experimental density ρ_m_ is lower than the expected density derived via classical SMR ρ_r_, see eq 17.

If ρ_m_ < ρ_r_, then, this means that, at the same blend composition, mass is constant and in accordance with eq 18 (1/*V*_m_ < 1/*V*_r_), meaning that *V*_m_ > *V*_r_. This phenomenon indicates Excess phenomena, as a matter of fact.

Volume excess phenomena with direct actuation on the increasing or decreasing of volume and in a specific volume expansion where *V*_m_ > *V*_r_ with ρ^E^ < 0 depend on the magnitude of intermolecular forces. Such forces are intrinsically electrostatic due to their nature that involves charge density along molecules and between molecules, being attractive in essence.

Intermolecular forces are supported by intermolecular bonds, which are classified as weak secondary bonds (<100 kJ mol^–1^). Any electron is transferred or shared in the interaction, and the phenomenon is based on the charge density.

The most frequent classification of secondary bonds allows us to consider the following terminology for the interactions contributing to intermolecular forces: van der Waals bonding and hydrogen bonding. A bond type of van der Waals: the term designs two types of interactions as is known: dipole–dipole interactions and Dispersion forces also called as London forces or Dispersion London forces. Dipole–Dipole interactions are correlated to polar molecules with a strength depending on the magnitude of the dipole moment of each molecule being operated by the order of 1/*r* ^3^. Taking into account the dipole moment of a molecule, it can induce a moment of dipole called induced dipole in a nonpolar molecule. Dispersion forces arise from transient dipole moments generated by fluctuations in the electronic charge density within a molecule, which in turn induce dipoles in neighboring molecules, leading to instantaneous dipole–induced dipole interactions. The dispersion forces are operating in all liquids being operational to short distances, 1/*r* ^6^. The number of atoms and the geometry of the molecule affect the strength of the dispersion force.

After the mixture underwent beyond the effects of molecular interaction, the volume of the mixture increased since molecules of both liquids can interact by dipole (biodiesel)–dipole induced (hydrocarbons) and dispersion forces reorganizing themselves and giving a new molecular volume.

According to the previous discussion of Figure 10a,b, item (i), the relation ρ_m_ < ρ_r_ indicates that the parameter ρ^E^ < 0, the excess density phenomenon negative represents an expansion in the volume of mixture that is not major effect attraction forces of type dipole (biodiesel)–dipole induced (hydrocarbons) and dispersion forces.

The parameter Excess molar volumes *V* ^E^ of the blends was derived using eqs 19–22, which has been widely accepted to describe excess molar volumes:^38,60–64^

19 $$V^{E}=V_{m}-V_{r}$$

where *V* is the molar volume parameter, superscript E means excess property, while subscript m is measured and r is the parameter derived by SMR, and *i* is a pure component. The analysis of eq 19 shows that *V*_m_ is equal to *V*_r_, and therefore, excess volume *V* ^E^ is equal to zero, as an ideal mixture.

20 $$V_{m}=\frac{1}{\rho_{m}}\left(\sum_{i=1}^{2}x_{i}M_{i}\right)$$

20a $$V_{m}=\frac{1}{\rho_{m}}\left(x_{1}M_{1}+x_{2}M_{2}\right)$$

where *x* is the molar fraction parameter, *M* is the molecular weight parameter, and ρ is the density parameter. Subscript m is measured, here assigned to the experimental data, and *i* represents the components biodiesel and petrodiesel, *i* = 1 and 2, respectively. The parameter *V*_r_ is given by eq 21:

21 $$V_{r}=\sum_{i=1}^{2}x_{i}V_{i}=\sum_{i=1}^{2}\frac{x_{i}M_{i}}{\rho_{i}}$$

21a $$V_{r}=x_{1}V_{1}+x_{2}V_{2}$$

In fact, the parameter *V*_r_ given by eq 21 describes volume evolution by a SMR (eq 21a). According to eqs 20, 21, and 21a, before applying, eq 19 is necessary to derive both parameters *x*_*i*_ and *M*_*i*_. The molecular weight *M*_1_ of diesel is considered as approximately 211.7 g mol^–1^.^65^ An estimated average molecular weight *M*_2_ for soybean/methyl-alcohol biodiesel (292.81 g mol^–1^) was determined based on the data presented in Table 3,^1^ according to eq 22:^66^

22 $$M_{2}=\sum_{j=1}^{15}f_{j}M_{j}$$

where *f* and *M* are the mass fractions and molecular weight, respectively. The subscript *j* varies from 1 to 15, meaning the set 15 methyl esters corresponding to the 15 fatty acids listed in Table 3, respectively. The four more abundant fatty acid methyl esters (FAMEs) were designed using Ascalaph designer software and are shown in Figure 11.

**Figure 11:**
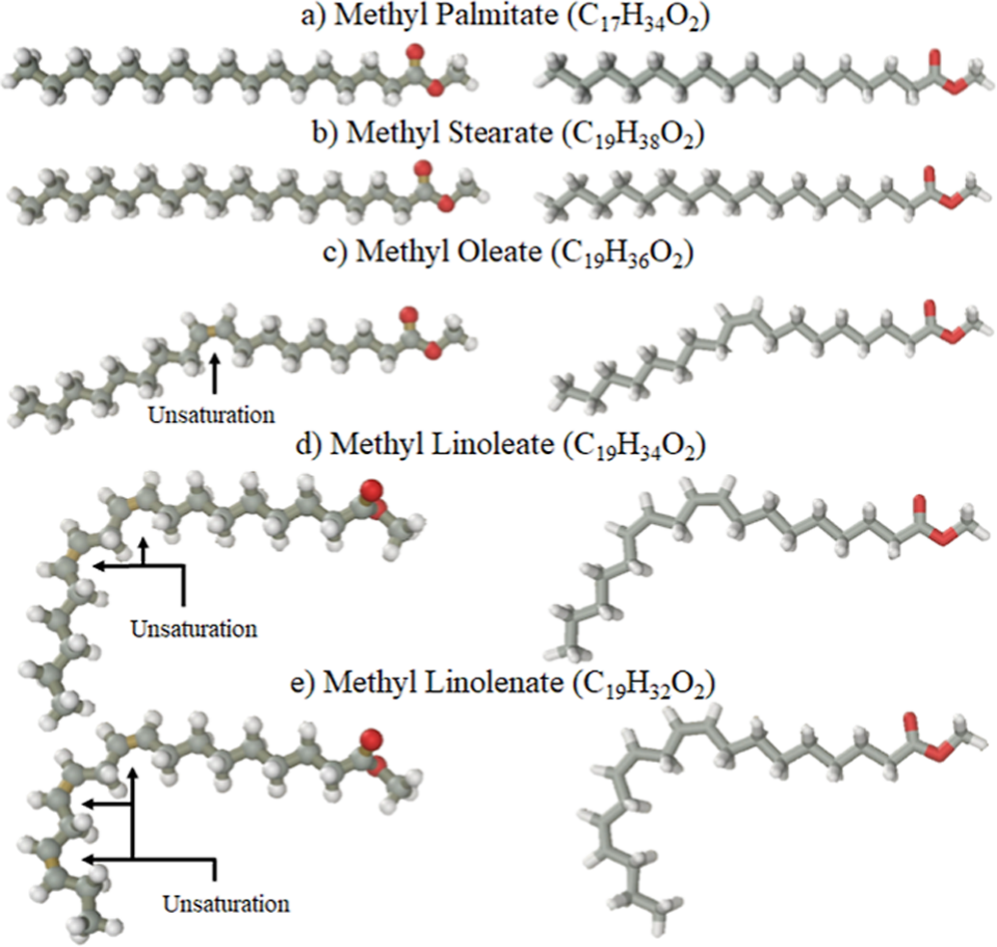
Model molecules of the five more abundant FAMEs present in biodiesel synthesized from soybean oil and methyl alcohol, ordered by the amount of unsaturation in each: (a) methyl palmitate, (b) methyl stearate, (c) methyl oleate, (d) methyl linoleate, and (e) methyl linolenate.

Each molecule was built, and then short optimization tool was run repeatedly until no modification was caused in the molecule’s structure. A fraction of FAMEs in biodiesel depends on the raw material used in the synthesis. Biodiesel has as major components such as the following molecules: methyl palmitate (C_17_H_34_O_2_), methyl stearate (C_19_H_38_O_2_), methyl oleate (C_19_H_36_O_2_), methyl linoleate (C_19_H_34_O_2_), and methyl linolenate (C_19_H_32_O_2_). Methyl ester molecule is linear only at saturated molecules (C=C double bonds are absent), as an example methyl palmitate (C_17_H_34_O_2_) and methyl stearate (C_19_H_38_O_2_), see Figure 11a,b.

As a whole, methyl esters are composed of saturated ester that exhibits a chain without the C=C double bond and monounsaturated ester with one C=C double bond in the chain. It is interesting to note that the presence of one C=C double bond seems to only rearrange the molecule in two linear parts of minor size, Figure 11c. However, being polyunsaturated with more than one C=C double bond at the chain, the molecule turns effectively nonlinear, see Figure 11d. The C=C double bond induces a significant break in the linearity. In fact, each double bond represents an additional break in the linearity of the carbon chain; see Figure 11e.

The eight more abundant molecules present in ultralow sulfur petrodiesel (ULSD) are as follows: decane (C_10_H_22_), dodecane (C_12_H_26_), tridecane (C_13_H_28_), tetradecano (C_14_H_30_), pentadecane (C_15_H_32_), hexadecane (C_16_H_34_), heptadecane (C_17_H_38_), and trimethylnaphthalene (C_13_H_14_) are shown in Figure 12.

**Figure 12:**
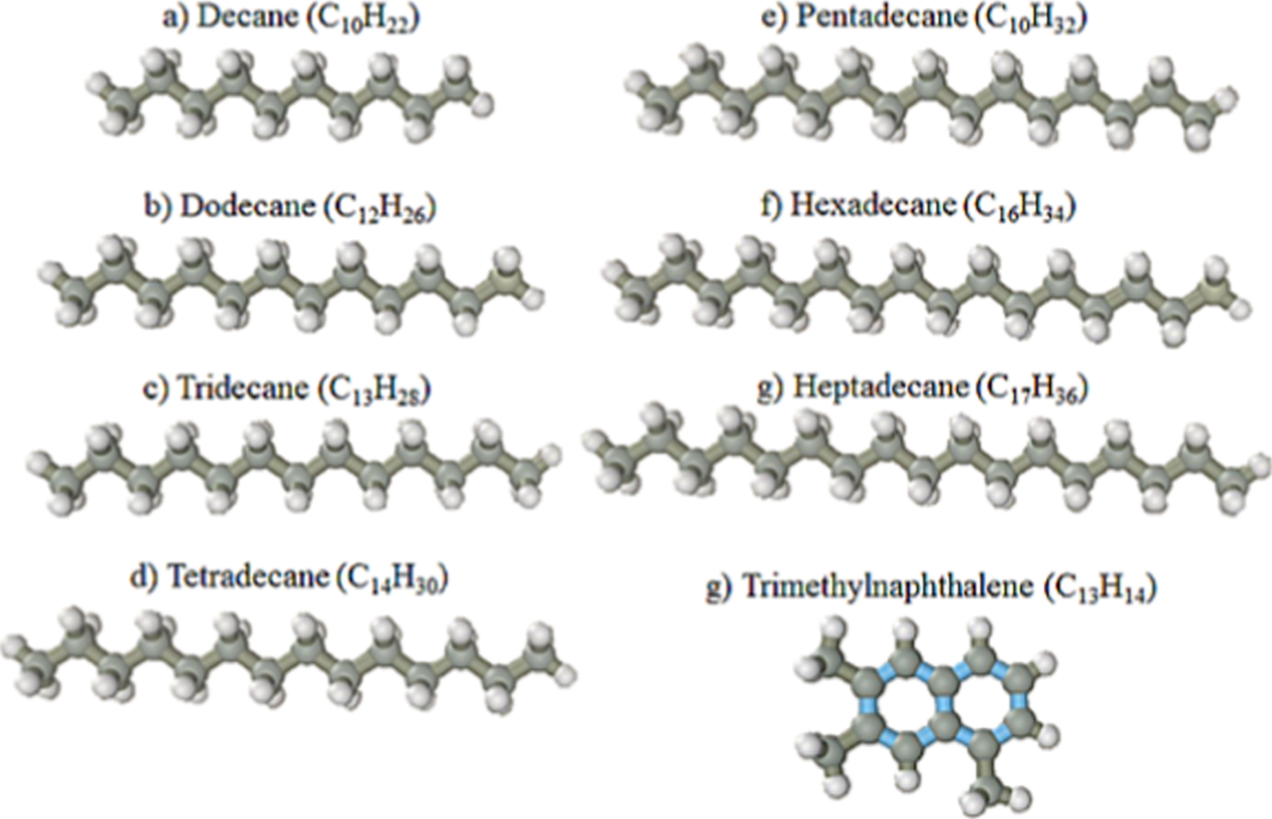
Model molecules of the eight more abundant compounds present in petrodiesel: (a) decane, (b) dodecane, (c) tridecane, (d) tetradecane, (e) pentadecane, (f) hexadecane, (g) heptadecane, and (h) trimethylnaphthalene.

All molecules mentioned in Figure 12 are linear, with C_13_H_14_ being a planar due to configuration.

However, Figure 13 shows molecules of the main sulfur compounds present in diesel, whose concentrations in the oil are reduced usually by hydrodesulfurization processes. These molecules comprise two groups: one for benzothiophenes and the other for dibenzothiophenes, both with possible alkyl substituents.

**Figure 13:**
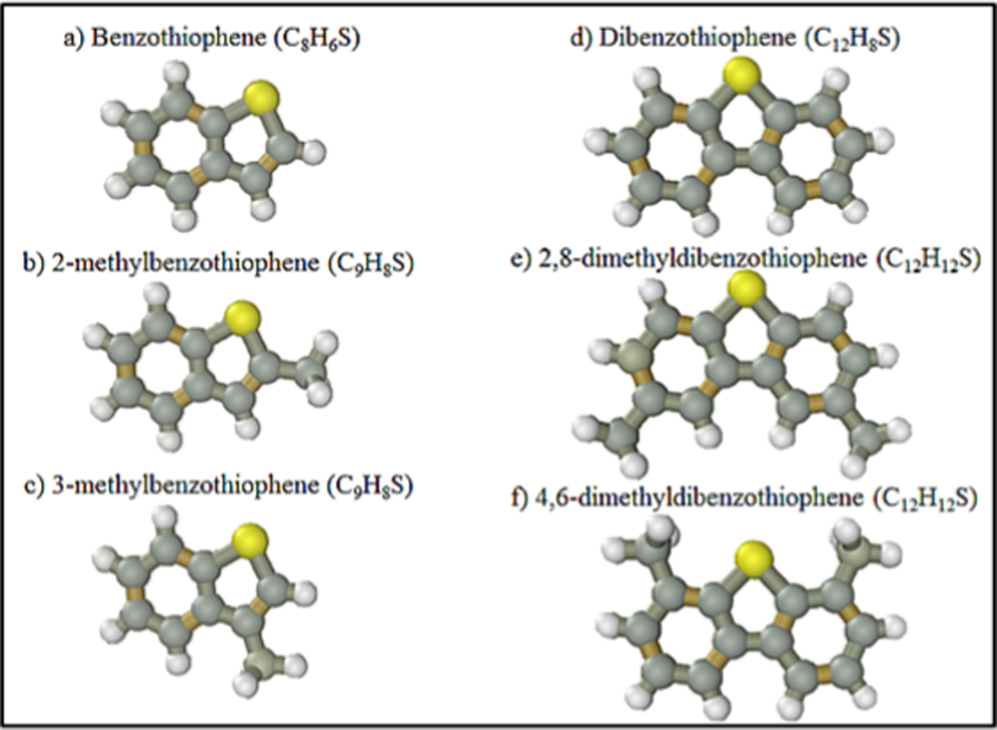
Model molecules of main groups of sulfur compounds present in petrodiesel: (a) benzothiophene, (b) 2-methylbenzothiophene, (c) 3-methylbenzothiophene, (d) dibenzothiophene, (e) 2,8-dimethyldibenzothiophene, and (f) 4,6-dimethyldibenzothiophene.

Molar fractions *x*_*i*_ were calculated from volume fractions by using eq 23:

23 $$x_{i}=\frac{\frac{\rho_{i}\phi_{i}}{M_{i}}}{\sum_{i=1}^{2}\frac{\rho_{i}\phi_{i}}{M_{i}}}$$

where **ϕ** represents the volumetric fraction parameter, *x* represents the molar fraction parameter, *M* represents the molecular weight parameter, and ρ represents the density parameter, while *i* represents a pure component, *i* = 1 or 2, then

23a $$x_{1}=\frac{\frac{\rho_{1}\phi_{1}}{M_{1}}}{\frac{\rho_{1}\phi_{1}}{M_{1}}+\frac{\rho_{2}\phi_{2}}{M_{2}}}$$

23b $$x_{2}=\frac{\frac{\rho_{2}\phi_{2}}{M_{2}}}{\frac{\rho_{1}\phi_{1}}{M_{1}}+\frac{\rho_{2}\phi_{2}}{M_{2}}}$$

The data obtained from eqs 19 to 23 are listed in Table 7. From the list of parameters in Table 7, blend biodiesel/petrodiesel exhibits an excess volume *V* ^E^ phenomenon.

**Table 7:** Molar Fraction (*x*_B_), Excess Thermal Conductivity (*k* ^E^), Thermal Conductivity by SMR (*k*_r_), Excess Molar Volume (*V* ^E^), Molar Volume of the Blend (*V*_m_) and the Molar Volume by SMR (*V*_r_) from Biodiesel/Diesel Blends

φ_B_ (%)	*x*_B_	*k* ^E^ (W mK^–1^)	*k*_r_ (W mK^–1^)	*V* ^E^ (cm^3^ mol^–1^)	*V*_m_ (cm^3^ mol^–1^)	*V*_r_ (cm^3^ mol^–1^)
0	0.000	0.000	0.129	0.00	256.70	256.70
10	0.079	–0.005	0.131	–0.02	267.74	264.74
20	0.162	–0.002	0.133	0.27	272.94	272.66
30	0.249	–0.004	0.134	0.29	280.77	280.48
40	0.340	–0.004	0.136	0.53	288.74	288.20
50	0.436	–0.002	0.138	1.06	296.88	295.82
60	0.537	–0.001	0.140	1.21	304.55	303.34
70	0.643	–0.002	0.1422	1.50	312.26	310.76
80	0.755	–0.003	0.143	1.54	319.61	318.07
90	0.874	–0.004	0.145	0.36	325.64	325.28
100	1.000	0.000	0.147	0.00	332.41	332.41

The differences between experimental data and prediction of values by the conventional SMR are further visualized using the relation between the parameters of molar-volume *V*_m_ and *V*_r_, described as excess molar volumes *V* ^E^, see eq 19, and can exhibit one of the following rules:

i *V* ^E^ < 0 or *V* ^E^ negative implies that *V*_m_ < *V*_r_ and ρ^E^ > 0. Negative values mean a volume contraction. The volume of the mixture is lower than the sum of the volume for each component.
ii *V* ^E^ > 0 or *V* ^E^ is positive implies that *V*_m_ > *V*_r_ and ρ^E^ < 0, rule (ii). Positive values indicate a volume expansion. The volume of the mixture is higher than the sum of the isolated components’ volumes.
iii *V*_m_ = *V*_r_, implying that *V* ^E^ = 0 and ρ^E^ = null. If *V* ^E^ = 0, the volume of the mixture is exactly the sum derived by the SMR.

According to Figure 10a,b, experimental data of density ρ_m_ and its expected valor derived by the SMR ρ_r_ do not match. Point by point, the experimental density ρ_m_ is lower than the expected density derived by means of classical SMR ρ_r_, see eq 17. The mass being constant ρ_m_ < ρ_r_, then, this means that at the same blend composition, and in accordance with eq 18, the 1/*V*_m_ < 1/*V*_r_ relation obeys *V*_m_ > *V*_r_, a volume expansion, as described in subitem (v). This phenomenon indicates that the blend underwent a complex formation with several types of molecular interaction actuation, which gave as a liquid results the volume expansion. The chemical interaction between molecules of both liquids can occur by dipole (biodiesel)–dipole induced (hydrocarbons) and dispersion forces reorganizing themselves and giving a new molecular volume. In a general way, a *V* ^E^ < 0 as described in subitem (i) can be assigned to the contribution of strong chemical interactions being further correlated to secondary chemical bonds involved in the liquid cohesion via hydrogen bonds, as an example.

Despite this, esters do not form hydrogen bonds with their molecules. In this sense, a dipole-induced–dipole-induced, dipole–dipole induced, dipole–dipole and hydrogen bond, as well as proton-acceptor composing possible types of chemical interactions, gives a contraction volume. Therefore, despite the effect of dipole (biodiesel)–dipole induced (hydrocarbons) and dispersion forces, which represent attraction forces to be present, a major molecular interaction seems to stem from another type of molecular interaction, see Figure 10c discussion. According to the previous discussion of Figure 10a, the relation ρ_m_ < ρ_r_ indicates ρ^E^ < 0, and the excess density phenomenon negative represents an expansion of mixture volume. According to Figure 10c, only the first blend ϕ_B_ equal to or at around 10% exhibits the parameter *V* ^E^ < 0.

For another fraction of biodiesel, rule (ii) states the parameter *V* ^E^ > 0, meaning that Expansion volume is operational, and the result of the contribution of physical interactions between molecules is also called a steric phenomenon.

The Excess volume *V* ^E^ parameter meant that the volume measurement of the mixture is different from the volume expected or theoretical volume, which should be a pondered sum of volumes of the components with a linear trend, see eq 21a. If there exists some kind of physical interaction between molecules, which involves the geometry of the molecule, a changing of the packing of molecules of the mixture occurs, the excess volume should be positive. The positive values of the excess volume, *V* ^E^ > 0, can be ascribed to a physical phenomenon of decreasing the molecule packing and expansion volume, which as a whole has a weak intensity. The negative values of the Excess volume, *V* ^E^ < 0, can be ascribed to chemical and structural phenomena, giving an increase of the molecule packing resulting in a volume contraction that as a whole exhibits a strong intensity.^14,67^ A *V* ^E^ < 0 due to contribution of structural events is correlated to optimization of interstitial volume and geometrical adjustment based on the distinct free molar volume and molar volume of the components of the mixture. Results shown in Figure 10b indicate that in biodiesel/petrodiesel blends, these secondary chemical bonds are not strong enough to overcome the difficulty packing between the long chain methyl esters from biodiesel and linear and cyclic carbons from petrodiesel. These results corroborate with the ones of Feyzi et al.,^10^ although the biodiesel used was made from sunflower oil. Another possible contribution of this positive excess volume is that the complete nonpolar molecules of diesel discontinue some weak but existing dipole–dipole interactions between ester molecules, which increases the average distance between the blend’s molecules.

In contrast, blends of diesel with coconut biodiesel led to negative values of *V* ^E^ according to Mesquita et al.^68^ This analysis is based on the maximum experimental excess-volume value *V^E^* = 1.5404 cm^3^ mol^–1^, which corresponds to the blend with 80% biodiesel v/v (*x*_B_ = 0.7554).

### 3.3 Thermal and Excess Thermal Properties Analysis

The phenomenon of thermal conductivity (λ) in liquids is complex. However, a nonmechanistic approach can be used to model the thermal conductivity phenomenon in biodiesel, ultralow sulfur petrodiesel, and its blends in a qualitatively way. Grossly, the magnitude of the thermal conductivity (λ) of liquids can be classified into two great groups. One group is known as polar liquids, and the other is known as nonpolar or organic liquids. Polar liquids exhibit a higher thermal conductivity (λ) from 0.2 up to 0.6 W mK^–1^, while organic liquids have significantly lower values of thermal conductivity ranging from 0.12 up to 0.2 W mK^–1^. Here, it is important to comment that cohesion in liquids is ascribed to an electrostatic interaction between molecules called secondary bonds, such as permanent dipoles, induced dipoles, and hydrogen bonds, all involving charge density instead of one electron. In polar liquids, the permanent moment of dipole and/or the hydrogen bonds^67^ is determined at the highest thermal conductivity level. Otherwise, complex organic liquids such as diesel are mixtures of hydrocarbons, which are maintained in a cohesion state via induced dipoles, specific instantaneous induced dipoles stemming from dispersion forces. As expected, the thermal conductivity (λ) of biodiesel synthesized by the methylic route is higher than that of ultralow sulfur petrodiesel. In part, this feature can be understood as follows. Biodiesel is composed of a set of methyl esters compounds, see Figure 11 discussion. Each type of ester has a segment of the molecule exhibiting a long carbon chain and a terminal methyl group called the charged head, which has a permanent dipole moment considered of small magnitude. In this sense, sometimes biodiesel is further considered a nonpolar molecule. As a matter of fact, some methyl ester compounds are linear molecules, with the long carbon chain with high polarizability resulting in a great molecular interaction with similar molecules and dissimilar linear molecules. In this way, as a function of the great number of carbons, instantaneous induced dipoles or induced dipoles also have significant magnitude, providing further liquid cohesion. This cohesion of the electrostatic nature shows strength proportional to the carbon number of chains and the linearity of molecules that guarantee the highest level of closeness between molecules.

The cohesion has its major intensity as a function of linearity and the closer degree of molecules characterizing the dispersion forces. The interaction can be considered to be of a degree similar to the dipolar interactions. Then, the intensity of dispersion forces can be modulated by changing the linearity of molecules, polarizability, the number of carbon atoms in the linear segments, and the average distance between molecules.

Experimental values of thermal conductivities (*k*_m_) as a function of the volumetric fraction of biodiesel ϕ_B_ and of biodiesel/petrodiesel blends are listed in Table 6. Figure 14 shows both thermal conductivity and excess thermal conductivity (*k* ^E^) parameters of the studied blends as a function of biodiesel volumetric fraction ϕ_B_. As expected, the thermal conductivity of the blends increases in a nonlinear manner as a function of biodiesel fraction in the blend.

**Figure 14:**
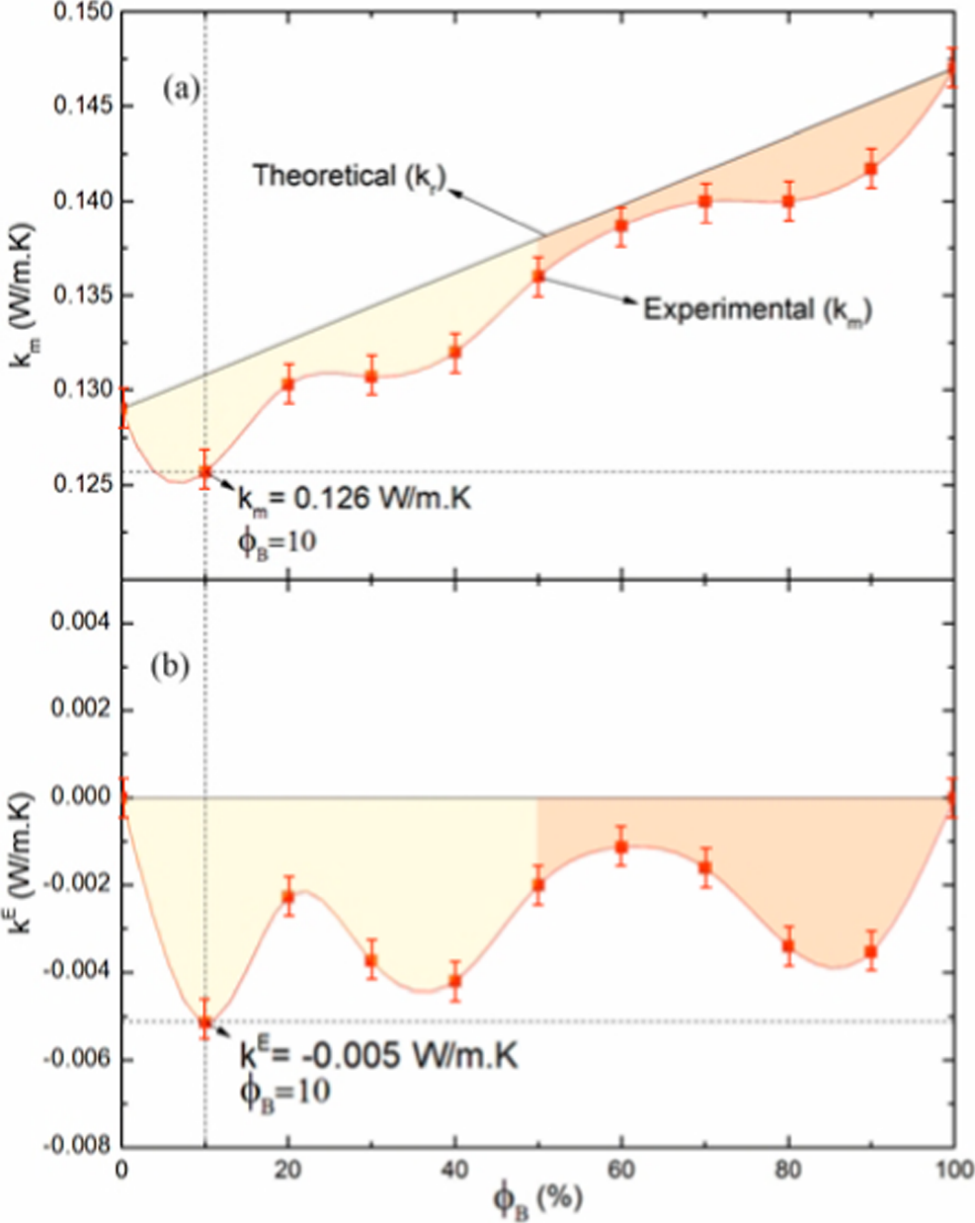
Plots of (a) thermal conductivity measured (*k*_m_) and (b) excess thermal conductivity (*k* ^E^) of biodiesel/petrodiesel blends versus biodiesel volumetric fraction ϕ_B_. The red dots represent the experimental data. The red line represents a cubic spline interpolation of the experimental data, and the black line represents (a) values calculated with SMR (*k*_r_) and (b) zero.

The evolution of experimental parameters of thermal conductivity (*k*_m_) as a function of the volumetric fraction ϕ_B_ of biodiesel is shown in Figure 14a; the red dots represent the experimental data. Points are connected only to auxiliary visual inspection of data changes. The black line represents theoretical thermal conductivity (*k*_r_) values derived from the SMR called linear thermal conductivity. Theoretical thermal conductivities (*k*_r_) of blends were derived by means of SMR by using the following eq 24:

24 $$k_{r}=\sum_{i=1}^{2}\phi_{i}k_{i}=\phi_{2}k_{1}+\phi_{2}k_{2}$$

where *k* represents the thermal conductivity parameter, ϕ represents the volumetric fraction parameter, *r* represents the SMR, and *i* represents the same component as before. Then, it is possible to derive excess thermal conductivity, *k* ^E^, by analogy with eq 19 from eq 25:

25 $$k^{E}=k_{m}-k_{r}$$

where superscript E indicates excess property, while subscript m represents measured.

Figure 14b shows changing of parameter excess thermal conductivity (*k* ^E^) of biodiesel/petrodiesel of low sulfur content blends S10, as a function of biodiesel volumetric fraction ϕ_B_. The red line connecting experimental points represents only a visual help of the evolution of the values. By definition, *k* ^E^ values of both biodiesel and petrodiesel have values equal to zero.

All values of excess thermal conductivity are negatives, meaning that composition to composition, the thermal conductivity (*k*_m_) is lower than the thermal conductivity (*k*_r_) values that represent values expected point by point. Further insight into this experimental behavior is attained from the discussion carried out in Section 3.2. In this sense, the clear increase of blend volume in a wide range of biodiesel volumetric fractions shows the development of molecules separation, without increases in an effective moment of induced dipoles. According to Figure 14b, there is a difference between both experimental and theoretical linear thermal conductivity given by eq 25, and such a difference is in accordance with the previous discussion based on the molecular interactions from the ATR-FTIR technique. Interactions between molecules provide the observed excess of thermal conductivity. The experimental values of thermal conductivity (*K*_m_) change between the values of petrodiesel of ultralow sulfur and the value of biodiesel. Also, the exhibited data set supports the existence of manifestation of an intermolecular interaction in the binary blends, being that both phenomena of molecular rearrangement by means of the physical phenomenon and chemical interaction are sufficient to change the thermal conductivity of blends. The magnitude of the changes in the thermal conductivity parameters is relatively small. As a matter of fact, the accuracy of thermal analyses is equal to 1%, and the values of *K* ^E^ are only of the order of 0.5%. This event suggests that changes in blends’ thermal properties are typical of small magnitude, as a function of biodiesel volumetric fraction ϕ_B_. Such features suggest that configurational aspects have strong participation in the blend mixture properties; in fact, the Steric phenomenon shows a natural barrier to dispersion forces and dipole–dipole-induced actuate effectively.

This feature was also investigated by ATR-FTIR, and volume excess exhibits a particular nature.

From Section 3.1 discussion, it is possible to observe an almost linear evolution of parameters such as area, amplitude, fwhm, and the center of the characteristic band positioned at around 1742 cm^–1^. Then, chemical and physical interactions are simultaneously operational, as a function of the presence of nonlinear molecules in both components of the blend. Furthermore, the existence of physical interactions called the steric effect exerted by nonlinear molecules can explain the nonideal (linear) behavior identified in the set of curves intensity of the band, the area of the band, and fwhm, as a function of biodiesel fraction. As a whole, experimental data about thermal conductivity can be analyzed as follows:

(i) *k* ^E^ < 0 or *k* ^E^ is negative implies that *K*_m_ < *K*_r_ with ρ^E^ < 0, see rule (ii) and *V* ^E^ > 0, see rule (v). Negative values indicate a decrease in thermal conductivity. The thermal conductivity of the blend is lower than the sum of thermal conductivity of each component of the blend.
(ii) *k* ^E^ > 0 or *k* ^E^ is positive implies that *K*_m_ > *K*_r_ with *V* ^E^ < 0 and ρ^E^ > 0. Positive values indicate an increase in thermal conductivity. The thermal conductivity of the blend is higher than the sum of thermal conductivity of each component.
(iii) *K*_m_ = *K*_r_, implying that *K* ^E^ = 0 and ρ^E^ = null. If *V* ^E^ = 0, the thermal conductivity of the blend is exactly the sum of the isolated components’ thermal conductivity.

Negative Excess thermal conductivity *k* ^E^ < 0 confirms the interaction between linear and nonlinear molecules of hydrocarbon (petrodiesel) and methyl esters (biodiesel), giving a decrease of packing (Excess volume) of molecules with direct effect under the thermal conductivity of the mixture. In this sense, excess thermal conductivity shows three minimum relatives measured at different blend compositions, meaning that distinct degrees of chemical and physical molecular interactions occur in the molecular clusters formed.

## 4 Conclusion

Soy biodiesel and petrodiesel, with an ultralow sulfur level of around 10 ppm/kg, as well as its binary blends based on the biodiesel fraction ϕ_Β_ equal to 10, 20, 30, 40, 50, 60, 70, 80, and 90% in vol were characterized via ATR mid-infrared, pycnometry, and thermal conductivity. Molecular interactions based on excess phenomena were successfully investigated. Both sets of molecules of biodiesel and petrodiesel underwent significant chemical and physical interactions. Such interactions occur via intermolecular phenomena based on both electrostatic and conformational molecule ways. In addition, dissimilar molecules with distinct carbon chains and molecules of nonlinear shape reach minor interactions further called as physical interactions. These types of interactions physically lead to the optimization of the structural arrangement of the blend with direct effect under distinct optimal molecular packing. Molecular geometry changes the auto-organization of molecules. Physical interactions also called the Steric phenomenon are composed of the major effect associated with the development and modulation of Excess properties. Further physical contributions of the Steric phenomenon are supported by the existence of Positive Excess volume and the development of a relative maximum in blends, as a function of the biodiesel fraction.

## Data Availability

Data are available throughout the manuscript.
